# Nickel isotopic evidence for late-stage accretion of Mercury-like differentiated planetary embryos

**DOI:** 10.1038/s41467-020-20525-1

**Published:** 2021-01-12

**Authors:** Shui-Jiong Wang, Wenzhong Wang, Jian-Ming Zhu, Zhongqing Wu, Jingao Liu, Guilin Han, Fang-Zhen Teng, Shichun Huang, Hongjie Wu, Yujian Wang, Guangliang Wu, Weihan Li

**Affiliations:** 1 State Key Laboratory of Geological Processes and Minerals Resources, China University of Geosciences, Beijing, 100083 China; 2 Laboratory of Seismology and Physics of Earth’s Interior, School of Earth and Space Sciences, University of Science and Technology of China, Hefei, Anhui 230026 China; 3 Department of Earth Sciences, University College London, London, WC1E 6BT UK; 4 CAS Center for Excellence in Comparative Planetology, USTC, Hefei, China; 5 Isotope Laboratory, Department of Earth and Space Science, University of Washington, Seattle, WA 98195 USA; 6 Department of Geoscience, University of Nevada, Las Vegas, NV 89154 USA

**Keywords:** Solid Earth sciences, Geochemistry

## Abstract

Earth’s habitability is closely tied to its late-stage accretion, during which impactors delivered the majority of life-essential volatiles. However, the nature of these final building blocks remains poorly constrained. Nickel (Ni) can be a useful tracer in characterizing this accretion as most Ni in the bulk silicate Earth (BSE) comes from the late-stage impactors. Here, we apply Ni stable isotope analysis to a large number of meteorites and terrestrial rocks, and find that the BSE has a lighter Ni isotopic composition compared to chondrites. Using first-principles calculations based on density functional theory, we show that core-mantle differentiation cannot produce the observed light Ni isotopic composition of the BSE. Rather, the sub-chondritic Ni isotopic signature was established during Earth’s late-stage accretion, probably through the Moon-forming giant impact. We propose that a highly reduced sulfide-rich, Mercury-like body, whose mantle is characterized by light Ni isotopic composition, collided with and merged into the proto-Earth during the Moon-forming giant impact, producing the sub-chondritic Ni isotopic signature of the BSE, while delivering sulfur and probably other volatiles to the Earth.

## Abstract (web summary)

Based on Nickel isotope analysis of meteorites and terrestrial rocks, the authors suggest that the Bulk Silicate Earth has a sub-chondritic Nickel isotope composition. This signature is thought to result from the impact and accretion of a Mercury-like impactor which originated from the innermost Solar System.

## Introduction

The Earth experienced a protracted accretion history over several tens up to 100 million years, which proceeded by the collision of numerous planetesimals and planetary embryos^1,2^. A fundamental assumption was that the Earth’s building blocks as a whole were compositionally similar to undifferentiated chondritic meteorites. Researchers have looked among different classes of chondrites for the closest representative of the accreting materials that formed Earth^3–6^. However, emerging evidence points to a mismatch in many crucial elemental and isotopic ratios between chondritic meteorites and the accessible Earth, arguing for the possible accretion of additional materials that are chemically and isotopically different from extant meteorite collections^7–11^. Constraining the nature of these building blocks of Earth is important, because they not only provide fundamental information on terrestrial planet formation, but also help understand how the Earth evolved into its current habitable status.

The late accretion stages, including the Moon-forming giant impact and the late veneer event, likely account for only <10% of Earth’s total mass^12^, but they represent a critical step for Earth to build its life-essential volatile budgets^13–20^. Dynamical models of Earth’s growth suggest that the late accretion stages were highly heterogeneous, consisting of a mixture of materials from two genetically distinct reservoirs in the Solar nebula^20,21^. One end-member may originate from the inner Solar system and contain a reduced, non-carbonaceous component that is probably ‘missing’ in known meteorites^11,20–24^. The other may be oxidized, carbonaceous chondrite-like material from the outer Solar system^17,19–21,25^. When these materials were added to Earth is still debated^19–27^. The carbonaceous chondrite-like materials are commonly thought to be the source of major volatiles in Earth^28,29^. Recent high pressure–temperature experiments on metal alloy-silicate partitioning of volatiles (e.g., carbon, sulfur, and nitrogen), however, suggest that Earth’s volatile abundance patterns could have been largely established by impact of a sulfur-rich, differentiated planetary body with minimal contributions from carbonaceous chondrite-like materials^15,16^. Due to the lack of proper meteorite proxies, the nature of late-stage impactors remains poorly known.

Nickel isotopic compositions of meteorites and terrestrial rocks may hold important clues. Nickel in the bulk silicate Earth (BSE) was mostly derived from late-stage impactors, as that from earlier stages was largely segregated into the core due to its moderately siderophile nature^23^. Models predict that ~95% of Ni in the BSE was derived from the last ~35% of mass that accreted to Earth^23,30^. Nickel is non-volatile and partitions compatibly into the mantle dominant phase – olivine – following accretion, so that the BSE can potentially capture the Ni isotopic signature of late-stage accreting materials.

**Fig. 1:**
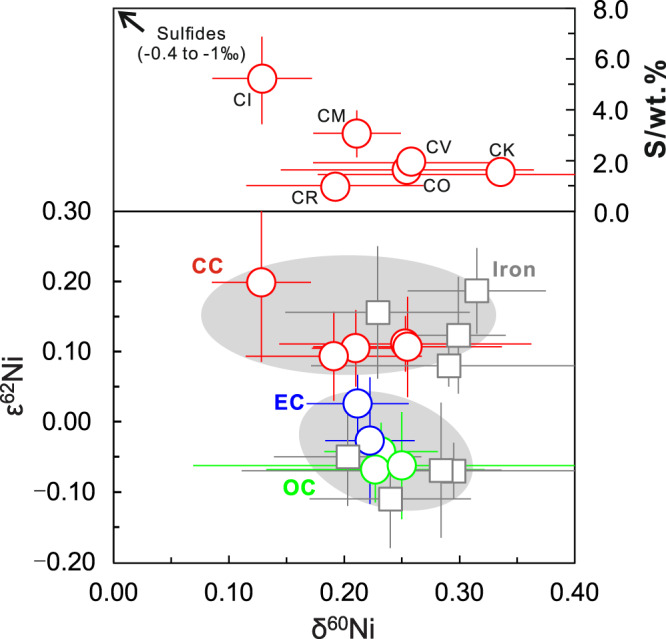
A summary of mass-independent and mass-dependent Ni isotopic variations in meteorites. The *ε*^62^Ni (*ε*^62^Ni = (^62/58^Ni_sample_/ ^62/58^Ni_SRM986_ − 1) × 10^6^ after internal normalization to ^61^Ni/^58^Ni), are from literature^31–33,35,36^, and the *δ*^60^Ni of chondrites and iron meteorites are from this study and literature^37–41,70^. The carbonaceous chondrites (CC), enstatite chondrites (EC), and ordinary chondrites (OC) have average *δ*^60^Ni values of 0.23 ± 0.14‰ (2 SD), 0.22 ± 0.02‰ (2 SD) and 0.24 ± 0.02‰ (2 SD), respectively. A roughly negative correlation between *δ*^60^Ni and sulfur abundance is observed in carbonaceous chondrites (upper panel), which may be caused by the presence of various abundances of sulfides with *δ*^60^Ni values as low as −1‰^41,42^. The gray areas represent the dichotomy between carbonaceous and non-carbonaceous meteorites^36^. The sulfur abundances are from ref. ^71^. Meteorite data from this study and literature are presented in Supplementary Table 2. Error bars represent 2 SD.

Nickel isotopic variations in meteorites have been well documented as shown in Fig. 1. Mass-independent nucleosynthetic Ni isotope anomalies arise from the heterogeneous distribution of presolar matters in the Solar protoplanetary disk, and thus trace the provenance of Earth’s building blocks. The nucleosynthetic anomalies are present in carbonaceous and ordinary chondrites, with enstatite chondrites largely within error of the BSE^23,31–35^, supporting the general idea that the late-stage accreting materials mainly originated from an enstatite-like source region in the inner Solar system^23^. Iron meteorites display similar anomalies, together with different groups of chondrites, forming a dichotomy between carbonaceous and non-carbonaceous meteorites as found in many other isotope systems (e.g., Mo, Cr, Ru, Ti)^36^. Mass-dependent isotopic variations stem from physico-chemical processes in the Solar nebula and on the planetary parent bodies. Nickel isotopic compositions of enstatite, ordinary, and most carbonaceous chondrites exhibit a common value, expressed as *δ*^60/58^Ni (the ^60^Ni/^58^Ni ratio in parts per thousand, relative to the SRM986 standard; *δ*^60/58^Ni = (^60/58^Ni_sample_ / ^60/58^Ni_SRM986_ −  1) × 1000), with an average of +0.23 ± 0.11‰ (2 SD, *n* = 34)^32,37–40^. The small isotopic variation observed in the carbonaceous chondrites most likely reflects the heterogeneous distribution of an isotopically light sulfide component^41,42^, supported by the roughly negative correlation between *δ*^60/58^Ni and sulfur content (Fig. 1). It is not surprising that iron meteorites have *δ*^60/58^Ni values within the ‘chondritic’ range (Fig. 1), because they represent fragments of the disrupted cores of planetary bodies, and dominate the Ni budget.

The Ni isotopic composition of present BSE is poorly constrained. An earlier report of a few ultramafic rocks yielded *δ*^60/58^Ni values indistinguishable from the chondritic average, and they concluded that the BSE has a chondritic Ni isotopic composition^39^. This conclusion is questioned in a recent study combining new and reported peridotite samples^40^, which suggested that the BSE has a *δ*^60/58^Ni ~0.1‰ lower than the chondrite average, a difference that was attributed to Earth’s core formation^40,43^. Central to this debate is the limited Ni isotope data for terrestrial silicate rocks and scant information on Ni isotope fractionation during igneous and core–mantle differentiations.

Here, we show that the BSE has a sub-chondritic Ni isotopic composition by applying Ni isotope analysis to meteorites and terrestrial rocks. Our first-principles calculations further suggest that the light Ni isotopic signature of the BSE is not a result of core–mantle differentiation. Rather, the signature was established during Earth’s late-stage accretion, via impact and accretion of a highly reduced, Mercury-like impactor that likely originated from the innermost Solar system.

## Results and discussion

### Non-chondritic Ni isotopic composition of the bulk silicate Earth

**Fig. 2:**
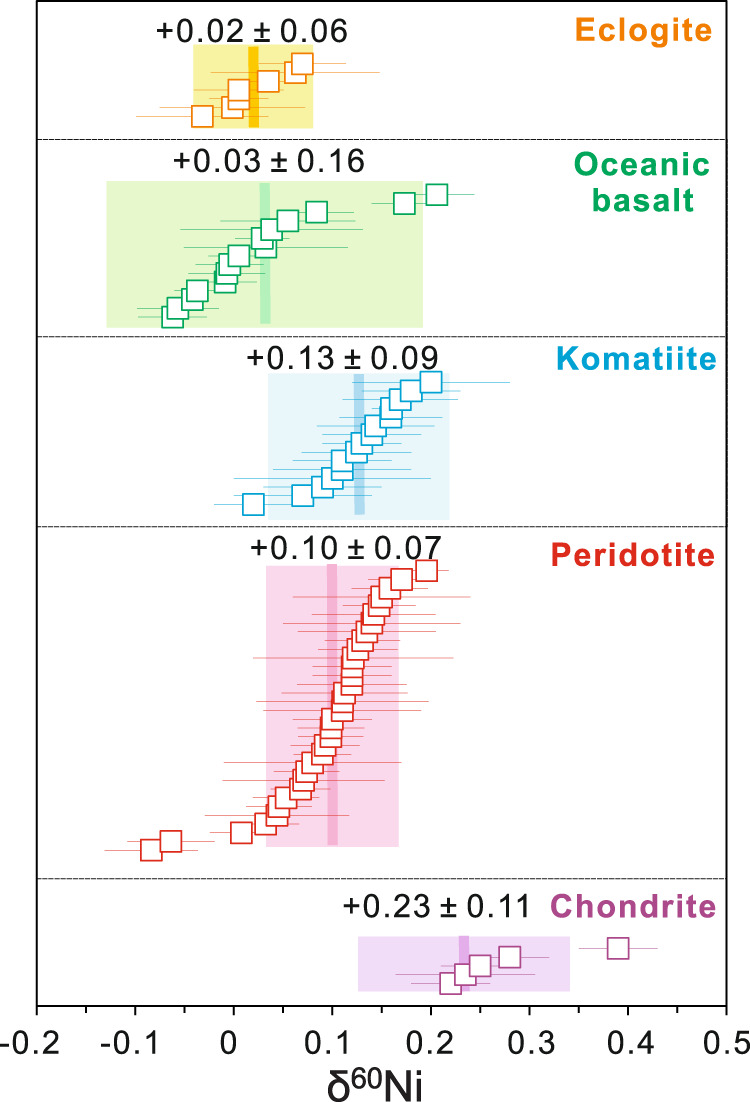
Mass-dependent Ni isotopic variations of terrestrial silicate rocks and chondrites from this study. The bands represent the average value for each sample category with 2 SD. The *δ*^60/58^Ni of five chondrite samples fall within the chondritic average defined by all published data (0.23 ± 0.11‰). The fertile and non-metasomatized peridotites with chemical compositions most close to the BSE^44^ have homogeneous Ni isotopic compositions, while those experienced secondary modification have relatively large variations. Therefore, only fertile, non-metasomatized peridotites are used for the average calculation (0.10 ± 0.07‰). Data for chondrites are presented in Supplementary Table 2; Data for peridotites, komatiites, oceanic basalts, and eclogites are presented in Supplementary Tables 4–6. Error bars represent 2 SD.

Our new high-precision, inter-laboratory analyses on 60 terrestrial silicate rocks demonstrate that the present BSE is unambiguously sub-chondritic. Fertile peridotites, whose major element compositions are closest to the Primitive Mantle (e.g., Mg^#^ = 89.6 ± 1.0; Al_2_O_3_ = 3.52 ± 0.60 wt.%)^44^, have *δ*^60/58^Ni values clustering tightly around +0.10 ± 0.07‰ (2 SD; *n* = 13, Fig. 2 and Supplementary Fig. 2). Peridotites overprinted by mantle metasomatism have Ni isotopic compositions shifted towards either heavier or lighter, but only to a limited degree (Supplementary Note 1). The melting products of mantle have similar or lighter Ni isotopic compositions compared to peridotites (Fig. 2). Komatiites formed by high-degree mantle melting (>45%) record an isotopic signature similar to the fertile peridotites (+0.13 ± 0.09‰, 2 SD, *n* = 15; Fig. 2). Oceanic basalts (OIBs and MORBs), which are produced by relatively low-degree melting (<25%), have Ni isotopic compositions slightly lighter than peridotites (Fig. 2; Student’s *t* -test; *p* < 0.001), with an average *δ*^60/58^Ni value of 0.03 ± 0.16‰ (2 SD, *n* = 15). Eclogites, formed from metamorphism of basalts, display a similar average *δ*^60/58^Ni value of 0.02 ± 0.06‰ (2 SD, *n* = 7; Fig. 2). Whether the difference between oceanic basalts and peridotites implies possible Ni isotope fractionation during partial melting or results from the limited dataset of oceanic basalts deserves further investigations. Nevertheless, the present BSE, as best represented by fertile peridotites reported in this study and literature^40^, has *δ*^60/58^Ni of +0.11 ± 0.06‰, lower than the chondritic average, +0.23 ± 0.11‰ (Student’s *t* -test, *p* « 0.001; Supplementary Note 1).

### First-principles calculations on Ni isotope fractionation during core formation

The sub-chondritic Ni isotopic composition of the BSE could have resulted from two possible processes: isotope fractionation associated with Earth’s differentiation, or the accretion of non-chondritic materials.

**Fig. 3:**
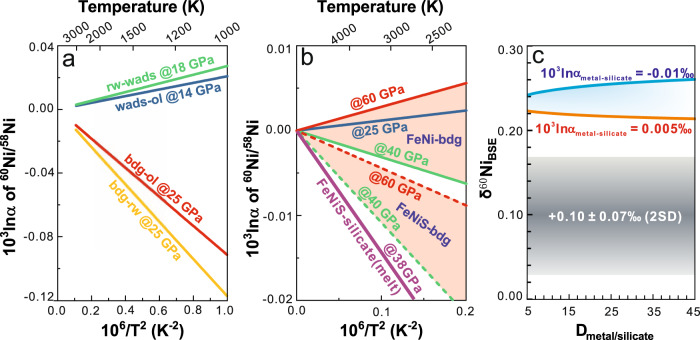
Equilibrium fractionation factors from first-principles calculations and the modeled Ni isotope fractionation during core–mantle differentiation. **a** Equilibrium fractionation factors (10^3^ln*α*) between olivine, wadsleyite, ringwoodite, and bridgmanite. **b** Equilibrium fractionation factors between Fe–Ni (S) alloy and bridgmanite, and between FeNiS melt (Fe_92_Ni_5_S_3_) and silicate melt (Mg_30_NiSi_32_O_96_). **c** *δ*^60^Ni_BSE_ vs. distribution coefficient of Ni between metal and silicate (*D*^Ni^_metal–__silicate_). If the bulk Earth (BE) has a chondritic Ni isotopic composition, the BSE δ^60^Ni after core formation can be evaluated using the Rayleigh fractionation equation: *δ*^60^Ni_BSE_ − *δ*^60^Ni_BE_ = Δ^60^Ni_metal–__silicate_ × ln*f*_BSE_, where *f*_BSE_ is the fraction of Ni in the BSE. The *f*_BSE_ can be calculated using the mass balance model: *f*_BSE_ = *M*_mantle_/(*M*_mantle_ + *D* × *M*_core_). The masses of the mantle and the core are 0.675 and 0.325, respectively. Given that the *D*^Ni^_metal–__silicate_ is likely <45^47^, core segregation induced Ni isotope difference between metal and silicate cannot account for the light Ni isotopic composition of the BSE. ol olivine, wads wadsleyite, rw ringwoodite, bdg bridgmanite, FeNi Fe–Ni alloy, FeNiS S bearing Fe–Ni alloy or melt.

The former hypothesis is examined using first-principles calculations on Ni isotope fractionation factors (10^3^ln*α* of ^60^Ni/^58^Ni) among Earth’s major Ni-bearing phases: olivine, wadsleyite, ringwoodite, bridgmanite, and Fe–Ni alloy. Limited differences in 10^3^ln*α* are found between olivine and wadsleyite/ringwoodite in the mantle transition zone and bridgmanite in the lower mantle (e.g., 10^3^ln*α* < 0.05‰ at 1500 K and <0.03‰ at 2000 K; Fig. 3a), which excludes the possibility of a hidden reservoir enriched in heavy Ni isotopes in the mantle. This lends credence to the use of accessible mantle and mantle-derived samples as representative of the present BSE Ni isotopic signature. Nickel isotope fractionation between Fe–Ni alloys and silicates (e.g., bridgmanite) under core-formation conditions is also negligible (*P* = 25–130 GPa; Fig. 3b and Supplementary Note 2). Notably, incorporation of sulfur into the Fe–Ni alloy slightly reduces the force constant of Ni, leading to the enrichment of light Ni isotopes in Fe–Ni alloys relative to the silicates (Fig. 3b).

To directly assess the equilibrium Ni isotope fractionation between silicate and metallic melts during core–mantle differentiation, we performed first-principles molecular dynamic simulations on melt phases of Fe_92_Ni_5_S_3_ and Mg_30_NiSi_32_O_96_ based on the density functional theory. The 10^3^ln*α* between Fe_92_Ni_5_S_3_ and Mg_30_NiSi_32_O_96_ melts is −0.011‰ at ~38 GPa and 3500 K (Fig. 3b; Supplementary Note 2), further confirming the conclusion based on crystals that core–mantle differentiation does not significantly fractionate Ni isotopes.

Two experimental studies investigated equilibrium Ni isotope fractionation between metal and silicate mineral or melt at low pressures (≤1.3 GPa) and temperatures (≤1623 K)^45,46^. Both studies predict limited Ni isotope fractionation under core-formation temperatures (<0.01‰ in terms of *δ*^60/58^Ni at *T* > 3000 K), a result that is consistent with our first-principles calculations at higher pressures, implying negligible pressure effect on silicate–metal Ni isotope fractionation. If the bulk Earth (BE) has a chondritic Ni isotopic composition, mass balance calculations using high-pressure metal–silicate Ni elemental partition coefficients^47^ and isotope fractionation factors obtained from our first-principles calculation demonstrate that core–mantle differentiation cannot explain the sub-chondritic Ni isotopic composition of the BSE (Fig. 3c).

Other possible events including evaporative loss, collision erosion, and core–mantle chemical diffusion can also be discounted as causes for the sub-chondritic Ni isotopic signature of BSE^40^. Evaporative loss of Ni is unlikely given the relatively refractory nature of Ni. In addition, kinetic isotope fractionation associated with evaporation would lead to a heavy BSE Ni isotopic composition, opposite to observations (Fig. 2). Collisional erosion during Earth’s formation preferentially removed early formed basaltic crust^48^. The terrestrial oceanic basalts have an average *δ*^60/58^Ni (0.03 ± 0.16‰; 2 SD) slightly lower than the BSE value, and thus collisional erosion cannot explain the sub-chondritic Ni isotopic composition of BSE. The presence of a Ni chemical gradient between Earth’s core and mantle may induce diffusive isotope fractionation, due to the faster diffusivity of light isotopes relative to heavy ones. A one-dimensional diffusion model shows that core–mantle chemical diffusion produces ~0.1‰ variation in the silicate part but is restricted to the lowermost two kilometers of the mantle on a time scale of 10 million years (Supplementary Note 3).

### The nature of late-stage accreting materials and its implications

Therefore, the Earth’s mantle must have accreted sub-chondritic materials during its growth. In the early stages, metal and silicate melts equilibrate completely in the magma ocean^49^, so that the proto-BSE likely has a low Ni concentration and a chondritic Ni isotopic composition. Because of the moderately siderophile nature of Ni^23^ and a possible disequilibrium scenario for the late-stage accretion^49,50^, the BSE’s sub-chondritic Ni isotopic signature was likely established in the late stages. The late veneer following the main growth stage added the last <0.5% of mass to Earth and contributed <5% of the Ni budget of the BSE^18^. Hence, it is unlikely to be the event that produced the sub-chondritic Ni isotopic composition of BSE. To account for the observed Ni isotopic value of the present BSE, the late-veneer material accreted to the BSE would have to have had extremely low *δ*^60/58^Ni of around −2.5‰ (Supplementary Fig. 8), a value that has not been found in any natural rocks, and would be inconsistent with the Ni isotopic composition of an average carbonaceous chondrite-like material for the late veneer^17,19,24,25^. The last significant stage of Earth’s accretion was the Moon-forming giant impact, contributing >20% of Ni budget of the BSE^49^. The Ni isotopic composition of the BSE could have been strongly influenced by the Moon-forming impactor^18,23^. Assuming a chondritic Ni isotopic composition for the proto-BSE as discussed above, mass balance calculation suggests that materials accreted to the proto-BSE have *δ*^60/58^Ni values as low as −0.35‰ (Supplementary Fig. 8). Accordingly, the Moon-forming impactor is unlikely to have a composition represented by chondrites.

**Fig. 4:**
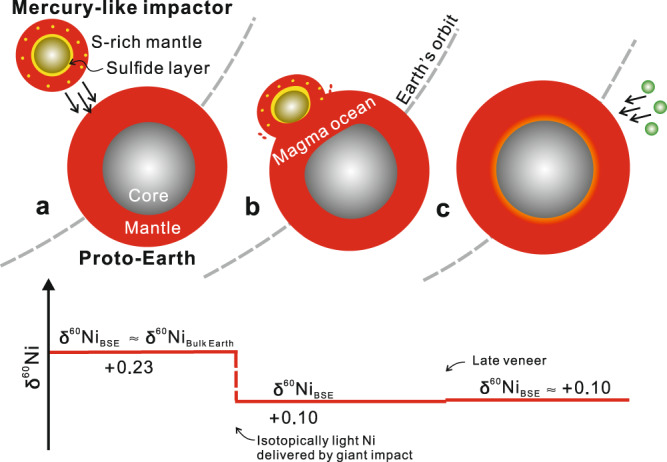
Cartoon showing the merger of a highly reduced, Mercury-like planetary body with the relatively more oxidizing proto-Earth, and schematic evolution of Ni isotopic composition of the BSE. **a** The small, proto-impactor from the highly reduced innermost region of the Solar system differentiated into a core, a sulfur-rich mantle, and likely a sulfide layer at the mantle–core boundary, because sulfur behaves more lithophile at low *f*O_2_ and pressure^58,59,61,62^. The impactor’s mantle likely had a sub-chondritic Ni isotopic composition due to the enrichment of sulfides. Differentiation on the large, proto-Earth partitioned sulfur dominantly into the core because sulfur is more siderophile at relatively high *f*O_2_ and pressure^58,59,61,62^. Therefore, the mantle of the proto-Earth had a Ni isotopic composition close to the chondritic value. **b** The Moon-forming giant impact would have completely melted the Earth, forming a planet-wide, more oxidizing magma ocean with high solubility of sulfur^60^. The sulfides in the impactor’s mantle were dissolved in the terrestrial magma ocean, producing a sub-chondritic Ni isotopic composition for the Earth’s mantle. **c** The late veneer following the main growth stage added carbonaceous chondrite-like materials to the Earth with limited effects on the Ni isotopic systematics of the BSE as discussed in the text.

Instead, we hypothesize that the sub-chondritic Ni isotopic composition of the BSE resulted from the impact and accretion of the sulfide-rich mantle of a highly reduced, differentiated planetary body. It has long been recognized that accretion of planetary embryos that were already differentiated into cores and mantles contributed significantly to the growth of Earth^51–53^. The Moon-forming impactor has been suggested to be a sulfur-rich, differentiated planetary body^15,16,27^; but uncertainties remain as to whether it is a highly reduced, Mercury-like impactor^23,24,27^, or a relatively more oxidized body^20,21,26^. Mercury is the most reduced planet in the inner Solar system and has an abnormally high abundance of sulfides in its mantle^54–56^, whereas oxidized planetary embryos have sulfur segregated into their cores^57^. This is because sulfur is highly siderophile at high oxygen fugacity (*f*O_2_) and partitions into the metallic core, but becomes lithophile and enters into the silicate melt as sulfide species under low *f*O_2_ (e.g., five units below the iron-wüstite buffer; IW-5)^58,59^. Magmatic sulfides are the only major Ni-bearing phases that are isotopically much lighter than silicates (*δ*^60/58^Ni_sulfide_ down to −1‰)^41^. Rocks with high sulfide/silicate ratios have light Ni isotopic compositions, which is most evident in magmatic Ni-sulfide deposits where the bulk *δ*^60/58^Ni values are negatively correlated with the sulfur content^41,42^. Therefore, when small planetary embryos (the proto-impactor) were formed in a sulfur-rich early Solar nebula and differentiated into core and mantle under highly reduced environment similar to the Mercury (mean IW-5.4)^58^, the mantle would be sulfur-rich and have a light Ni isotopic composition (Fig. 4). By contrast, in the large proto-Earth, core–mantle differentiation proceeded under much higher pressure and likely more oxidizing condition (>IW-3; Fig. 4)^60^, in which sulfur behaves as a siderophile element^61,62^, leading to a sulfur-poor mantle. In this case, limited silicate–metal Ni isotope fractionation is expected (see discussion above), and thus the proto-Earth mantle likely has a chondritic Ni isotopic composition. During the Moon-forming impact, the impactor’s core merged directly into the proto-Earth’s core due to its limited emulsification, while the remaining parts of the impactor were incorporated into the Earth’s mantle^49,50^. The Mercury-like impactor’s sulfur-rich mantle would have been completely dissolved in the planet-wide, more oxidizing terrestrial magma ocean^63^, and produced the sub-chondritic Ni isotopic signature of Earth’s mantle (Fig. 4).

Our findings imply that, instead of an outer Solar system origin^20,21^, the Moon-forming impactor may represent a ‘missing’ Earth’s building component originated from a highly reduced reservoir in the inner Solar system. While the absence of nucleosynthetic Ni isotopic anomalies in enstatite chondrites is consistent with an inner Solar system provenance for the Moon-forming impactor^23^, the sub-chondritic mass-dependent Ni isotopic composition of the BSE further points towards a sulfide-rich, Mercury-like impactor, likely from closer to the Sun. This is in accordance with variations in nucleosynthetic isotope anomalies of other elements in meteorites, suggesting that the Earth possesses the most s-process enriched materials from the inner Solar system^11,22,24,64^. A most recent study found s-process enriched ruthenium (Ru) isotopic signatures in Eoarchean rocks, supporting the idea that the pre-late veneer Earth incorporated building materials from the innermost region of the Solar system, most likely through the Moon-forming giant impact^24^. The impactor might be sulfur-rich, such that the highly siderophile Ru was partially retained in the mantle without being completely extracted to the core. Later addition of a carbonaceous chondrite-like late veneer with s-process Ru deficits from the outer Solar system ultimately built up the modern mantle Ru isotopic composition^24^. The proposed late-stage accretion of the highly reduced, Mercury-like planetary body may not only explain the broad geochemical similarity between Earth and Moon^27^, but also account for the volatile abundance patterns in the BSE^16^. Our study highlights the importance of inner planets, e.g., Mercury and Venus, in searching for the Earth’s ‘missing’ building blocks that are not present in extant meteorite collections. Future studies on achondrites from the inner Solar system and samples from Venus and Mercury as well as experimental work will shed more light on these issues.

## Methods

### Nickel isotope analyses

We undertook an inter-laboratory comparison of geological reference materials using different analytical protocols in two labs: Indiana University (IU) and China University of Geosciences, Beijing (CUGB). Despite different double spike solutions and different column chemistry methods, the results of standards from two labs agree with each other within analytical uncertainty (Supplementary Table 1). A comparison of our data with published values is shown in Supplementary Fig. 1.

#### Ni isotope analyses at IU:

Sample powders were digested in a mixture of distilled HF + HNO_3_ + HCl. After complete dissolution, aliquots of sample solutions containing 1.5 μg Ni were spiked with a ^61^Ni –^62^Ni double spike to reach an optimal spike–sample ratio of 64:36. The mixtures were refluxed on a hotplate to ensure sample–spike equilibration before column chemistry. Separation of Ni from the matrices was achieved using a three-stage, cation exchange chromatography procedure using Bio-Rad 200–400 mesh AG 50W-X8 resin. Briefly, the first column applies mixture of 20% 10 M HCl and 80% acetone to separate Ni from Fe, Mn, and Cr. The second column uses 15% 10 M HCl and 85% acetic acid to separate Ni from elements such as Mg, Al, Ca, and Ti, and the last column further purifies Ni using 0.9 M HNO_3_ to remove Na and K. The Ni isotopic ratios were measured using *Nu* Plasma II MC-ICPMS at Indiana University.

#### Ni isotope analyses at CUGB:

Sample powders were dissolved and spiked follow the same protocol at IU, but with a different ^61^Ni–^62^Ni spike solution to obtain optimal ratio of ^62^Ni_spike_/^58^Ni_sample_ = 1.3. The spike–sample solution was then passed through four-stage column chemistry. The step 1 column uses AG 50W-X8 and AG 1-X8 resins to separate Ni from Fe and Ca. Step 2 column uses AG 50W-X8 resin to separate Ni from Mg, Ti, and Al in a media of 0.15 M HNO_3_ and 4 M HF. A third column uses 0.5 M HCl containing 95% acetone to remove Mn; and the last column further separates Ni from the residual matrix using 0.5 M HCl + 95% acetone + 0.1 M DMG. The Ni isotopic ratios were determined on *Nu* Plasma III MC-ICPMS at the Laboratory of Surficial Environmental Geochemistry, Institute of Earth Sciences, China University of Geosciences (Beijing).

### First-principles calculations

Ab initio calculations were performed using the software “Quantum Espresso”^65^, which is based on the density functional theory (DFT), plane wave, and pseudopotentials. The generalized gradient approximation (GGA) was adopted to describe the exchange-correlation functional. The pseudopotential for magnesium was generated using von Barth and Car’s methods with a cutoff radius 2.5 Bohr. The electron configurations are 3s^2^3p^0^, 3s^1^3p^1^, 3s^1^3p^0.5^3d^0.5^, 3s^1^3p^0.5^, and 3s^1^3d^1^ with decreasing weights of 1.5, 0.6, 0.3, 0.3, and 0.2, respectively. The pseudopotentials for nickel﻿, silicon, and oxygen were generated by the method in Troullier and Martins^66^. The cutoff radius are 1.45 Bohr with the electron configuration of 2s^2^2p^4^ for oxygen and 1.47 Bohr with the electron configuration of 3s^2^3p^4^3d^0^ for silicon. The cutoff radius for nickel is 2.1 Bohr with the electron configuration of 4s^2^3d^8^4p^0^. The pseudopotential for Fe was generated using the Vanderbilt method^67^ with a valence configuration of 3s^2^3p^6^3d^6.5^4s^1^4p^0^ and a cutoff radius of 2.0 Bohr for Fe.

We first optimized all crystal structures of Ni-bearing minerals using the variable cell shape molecular dynamics method^68^ with different *k*-point grids dependent on the sizes of unit cells (Supplementary Table 7). The energy cutoff for plane wave and charge density are set to 70 Ry and 700 Ry, respectively. ﻿The residual forces converge within 10^−4^ Ry/Bohr. After the relaxed structures were obtained, we then calculated vibrational frequencies using the finite displacement method as implemented in the open-source code PHONOPY^69^. Consequently, the ﻿reduced partition function ratios *β* of ^60^Ni/^58^Ni for all phases can be calculated from the equation: $\beta _A = \frac{{Q_h}}{{Q_l}} = \mathop {\prod}\nolimits_i^{3N} {\frac{{u_{ih}}}{{u_{il}}}\frac{{e^{ - \frac{1}{2}u_{ih}}}}{{1 - e^{ - u_{ih}}}}\frac{{1 - e^{ - u_{il}}}}{{e^{ - \frac{1}{2}u_{il}}}}}$.

In order to directly estimate the equilibrium Ni isotope fractionation between silicate and metallic melts, we conducted first-principles molecular dynamics (FPMD) simulations on Mg_30_Ni_2_Si_32_O_96_ and Fe_92_Ni_5_S_3_ melts based on the DFT using the Vienna ab initio simulation package (VASP). The GGA was adopted for the exchange-correlation functional and the projector-augmented-wave (PAW) pseudopotentials were used. The energy cutoff for the plane wave was 600 eV. The Brillouin zone summations over the electronic states were performed at gamma point. The FPMD simulations were performed in the NVT thermodynamic ensemble with a fixed temperature of 3000 K and the Nosé thermostat was used. The time step was set to be 1 fs, and the total running time is up to 60 ps. The initial liquid configurations were prepared by conducting simulations on the structures at 6000 K. The cell parameter of the cubic box is 11.15 Å for Mg_30_Ni_2_Si_32_O_96_ silicate melt and 10.05 Å for Fe_92_Ni_5_S_3_ melt. The simulated statistical pressures of Mg_30_Ni_2_Si_32_O_96_ and Fe_92_Ni_5_S_3_ are 37.6 GPa and 38.5 GPa at 3000 K (Supplementary Fig. 5), respectively. After equilibration, we extracted 66 snapshots from the FPMD trajectory every 200 steps and only optimized the atomic positions of Ni with fixed cubic boxes. This strategy makes the Ni atoms in each snapshot are at  the local equilibrium positions. Then we estimated the force constant matrix of Ni atoms in all snapshots using the small displacement method based on the harmonic approximation. The force constants <F > of the Ni atom in Mg_30_Ni_2_Si_32_O_96_ and Fe_92_Ni_5_S_3_ melts are the cumulative averages in the time domain. According to the high-temperature approximation of the Urey equation, it can be written as: $\beta=1+(\frac{{1}}{{m_l}}-\frac{{1}}{{m_h}})\frac{{\hslash}^2}{{8}{{k_{B}}^{2}}{{T}^2}}<{F}>$

where *m*_l_ and *m*_h_ are the masses of light and heavy isotopes, respectively. The equilibrium Ni isotope fractionation between Mg_30_Ni_2_Si_32_O_96_ and Fe_92_Ni_5_S_3_ melts can be derived from the <F> difference between these two melts. The resulting <F> of Ni in Mg_30_Ni_2_Si_32_O_96_ and Fe_92_Ni_5_S_3_ melts at ~38 GPa are 272.1 ± 7.5 and 214.4 ± 2.8 N/m (Supplementary Fig. 6), respectively. Therefore, the 10^3^ln*α* between Mg_30_Ni_2_Si_32_O_96_ and Fe_92_Ni_5_S_3_ melts is 0.016 ± 0.003 ‰ at 3000 K and 0.011 ± 0.002 ‰ at 3500 K.

## Supplementary information

Supplementary Information

## Data Availability

All data in this study are included in the supplementary information files and are available from the corresponding author.
