# Supplementary material for: Nickel isotopic evidence for late-stage accretion of Mercury-like differentiated planetary embryos
Source: Nat Commun. 2021 Jan 12;12:294. doi: 10.1038/s41467-020-20525-1 (PMC7803775; doi:10.1038/s41467-020-20525-1)
Supplement: Supplementary file 1 — Supplementary Information [file 41467_2020_20525_MOESM1_ESM.docx]

Supplementary Materials for

**Nickel isotopic evidence for late-stage accretion of Mercury-like differentiated planetary embryos**

Shui-Jiong Wang^1*^, Wenzhong Wang^2,3,4^, Jian-Ming Zhu^1^, Zhongqing Wu^2,4^, Jingao Liu^1^, Guilin Han^1^, Fang-Zhen Teng^5^, Shichun Huang^6^, Hongjie Wu^1^, Yujian Wang^1^, Guangliang Wu^1^, Weihan Li^1^

^1^ State Key Laboratory of Geological Processes and Minerals Resources, China University of Geosciences, Beijing 100083, China

^2^ Laboratory of Seismology and Physics of Earth’s Interior, School of Earth and Space Sciences, University of Science and Technology of China, Hefei, Anhui 230026, China

^3^ Department of Earth Sciences, University College London WC1E 6BT, UK

^4^ CAS Center for Excellence in Comparative Planetology, Hefei, China

^5^ Isotope Laboratory, Department of Earth and Space Science, University of Washington, Seattle 98195, USA

^6^ Department of Geoscience, University of Nevada, Las Vegas 89154, USA

Correspondence to: [wsj@cugb.edu.cn](mailto:wsj@cugb.edu.cn)

**Supplementary Note 1: samples and materials**

***Chondrite samples***: We analyzed a representative selection of chondrites (Supplementary Table 2). The chondrites consist of three carbonaceous chondrites (Allende CV3, Karoonda CK4 and Kaba CV3), an ordinary chondrite (Chainpur LL3.4), and an enstatite chondrite (Kota-Kota EH3). The δ^60^Ni values vary from +0.22 to +0.39‰, falling in the chondritic range defined by previous studies (Supplementary Table 2). Integrating our data with published mass-dependent Ni isotope value of bulk chondrites determined using double-spike technique (Supplementary Table 2), the average δ^60^Ni values of the three groups are estimated to be +0.23 ± 0.14‰ for carbonaceous chondrite, +0.22 ± 0.02‰ for enstatite chondrite, and +0.24 ± 0.02‰ for ordinary chondrite. No significant difference in Ni isotope compositions exists among the three main groups of chondrites.

In order to constrain the Ni isotope composition of the Bulk Silicate Earth (BSE), terrestrial rocks including peridotite, komatiite, middle ocean ridge basalt (MORB), oceanic island basalt (OIB) and eclogites are analyzed. All of these samples have been well studied. Detail information on geological background, mineralogy and geochemistry can be found in Supplementary Table 3. Nickel isotope compositions of the samples analyzed in this study are given in Supplementary Tables 4-6.

***Peridotites***: Only two studies to date have systematically reported Ni isotope compositions of peridotites, and yielded different results (Supplementary Table 4). Gall et al.^1^ analyzed Ni isotope compositions of a suite of depleted peridotites (with Mg# varying from 91.2 to 93.1) from Tanzania and Kaapvaal cratons, and reported an average δ^60^Ni of +0.22 ± 0.08‰. Klaver et al.^2^ selected a suites of relatively fertile to moderately melt-depleted orogenic and cratonic peridotites (with Mg# varies between 88.6 and 91.7). The average δ^60^Ni value is +0.115 ± 0.011‰, which is lower than that of Gall et al.^1^.

Our new inter-laboratory analyses on 33 well-characterized peridotite samples from North China Craton and central-western Pyrenees suggest that the Ni isotope composition of the accessible Earth’s mantle is unambiguously sub-chondritic. The peridotites compositionally range from fertile to moderately refractory, with Al_2_O_3_ varying from 0.88wt% to 4.26wt% and Mg# from 88.3 to 91.5, none of which are as high as 93.06 as reported in Gall et al.^1^. Fertile, non-metasomatized peridotites, whose major elemental compositions are closest to the BSE (*e.g*., Mg^#^ = 89.6 ± 1.0; Al_2_O_3_ = 3.52 ± 0.60 %)^3^, have δ^60/58^Ni values clustering tightly around +0.10 ± 0.07‰ (2SD) (Supplementary Figure 2). This value may represent the best robust estimate of the BSE Ni isotope composition which is statistically lighter than the chondritic average (Student *t*-test; *p* << 0.001).

Mantle secondary modification could only shift the Ni isotope composition of peridotites towards either heavier or lighter values to a very limited degree (Supplementary Table 4 and Figure 2). Samples suffered from modal metasomatism as evidenced by the presence of amphibole and phlogopite display relatively large Ni isotopic variations, whereas fertile peridotites overprinted by cryptic metasomatism almost have similar Ni isotope composition to the non-metasomatized ones.

***Komatiites***: Komatiites are high-MgO volcanic rocks that are formed by high-temperature, high-degree of melting (>45%) within a mantle plume, likely preventing isotope fractionation from their mantle sources. Therefore, classic komatiites with ages ranging from 3.5-2.7 Ga are used to complement the fertile peridotites to place constraints on the Ni isotope composition of the mantle.

Two studies so far reported the Ni isotope compositions of komatiites (Supplementary Table 5). Gall et al.^1^ first carried out Ni isotopic analyses on the komatiites from Gorgona islands, Colombia. The five Gorgona komatiites give an average δ^60^Ni value of +0.21 ± 0.09‰. However, the Gorgona komatiites are the only documented Phanerozoic (~90 Ma) komatiites on Earth and are chemically distinct from typical Archean-Proterozoic komatiites, notably in terms of Ni^4^. The olivine phenocrysts in Gorgona komatiites have ‘excess Ni’ at a given Mg-number, which are about 20% higher than the olivines crystallized from primary magmas of fertile peridotites and those from other komatiite suites^4^. The ‘excess Ni’ is in accordance with the high ^3^He/^4^He ratio and ^186^Os anomaly determined in Gorgona komatiites, and was interpreted as resulted from origination from a mantle source that has interacted with the Earth’s high-Ni outer core^4^. Therefore, the Gorgona komatiites are not the best representative of the primitive mantle composition. In our previous paper^5^, we reported Ni isotope compositions of 11 Barberton-type komatiite samples from Komati, Hooggenoeg, and Sandspruit in South Africa. In this study, we analyzed four Munro-type komatiites from Canada. The spinifex structures are well developed and relatively fresh. The results show that both Barberton- and Munro-type komatiites have similar Ni isotope compositions, with the average δ^60^Ni value (+0.13 ± 0.09‰, 2SD) similar to that defined by fertile peridotites.

***Oceanic basalts***: Middle ocean ridge basalts (MORBs) and ocean island basalts (OIBs) represent mantle-derived oceanic basalts from relatively low degree melting (<25%). Neither have been systemically studied in Ni isotopes. Cameron et al.^6^ first reported three MORBs from East Pacific Rise. The δ^60^Ni values vary from +0.01 to +0.24‰. Gueguen et al.^7^ analyzed five oceanic basalts from IODP site 1149. Despite variable alteration, the five MORBs yield relatively homogenous Ni isotope compositions with the average δ^60^Ni of +0.03 ± 0.10‰ (2SD). In this study, we measured Ni isotope compositions of nine MORBs from East Pacific Rise, Central Indian Ridge, and North Atlantic Ridge (Supplementary Table 5). The average δ^60^Ni (+0.04 ± 0.18‰; 2SD) is similar to that reported by Gueguen et al.^7^. Prior to the present study, the only information on Ni isotope composition of OIBs is gained from two USGS standards (BHVO-1 and BHVO-2) and one basalt from Loihi, Hawaii, all of which yield δ^60^Ni values that are around 0.04‰^7^. We analyzed Ni isotope compositions for six OIBs from the Kilauea Iki lava lake in Hawaii (Supplementary Table 5), and obtained an average δ^60^Ni value of +0.01 ± 0.12‰ (2SD). Overall, Ni isotope compositions of oceanic basalts are statistically lighter than the fertile peridotites (Student *t*-test; p < 0.001), with an average δ^60/58^Ni value of 0.03 ± 0.16‰ (2SD), suggesting that partial melting may drive resolvable Ni isotope fractionations. Rapid consumption and relatively large contributions of isotopically light sulfides^7^ to melts during low-degree mantle melting could potentially produce the difference, but the detail mechanism deserves further investigations.

***Eclogites***: Basaltic rocks represent the major Ni-rich component in the crust. When recycled into mantle through crustal subduction, they are metamorphosed to eclogites. We analyzed seven eclogites including continental eclogites from Dabie orogen and oceanic eclogites from the western Tianshan, China. The eclogites yield an average δ^60^Ni of +0.02 ± 0.06‰ (2SD), indistinguishable from the oceanic basalts (Supplementary Table 6). Therefore, recycling of Ni-rich crustal components into the mantle cannot cause Ni isotope heterogeneity of the mantle, neither can they explain the Ni isotopic difference between oceanic basalts and fertile peridotites.

**Supplementary Note 2: results of theoretical calculations**

**Equilibrium isotope fractionation**

The mass-dependent equilibrium isotope fractionation arises from the isotopic substitution that induces a difference in vibrational frequency properties^8^. Following Richet et al.^9^, the ﻿reduced partition function ratio β_A_ of the element X in phase A, which represents the equilibrium X isotope fractionation factor between the phase A and an ideal atomic gas, can be written within the harmonic approximation as:

$\beta_{A}=\frac{Q_{h}}{Q_{l}}=\prod_{i}^{3N} \frac{u_{ih}}{u_{il}}\frac{e^{-\frac{1}{2}u_{ih}}}{1-e^{-u_{ih}}}\frac{1-e^{-u_{il}}}{e^{-\frac{1}{2}u_{il}}}$ (1)

where *h* and *l* represent the heavy and light isotopes, respectively, running index *i* refers to the *i*^th^ vibrational frequency, and *N* is the number of atoms in the unit cell. *Q_h_* and *Q_l_* are the vibrational partition functions for the heavy and light isotopes, respectively. $u_{ih}$ and $u_{il}$ are defined as:

$u_{ih or il}=\hbar\omega_{ih or il}/k_{B}T$ (2)

where $\hbar$ and $k_{B}$ are the reduced Planck and Boltzmann constants, respectively, *T* is temperature in Kelvin, and $\omega_{ih or il}$ is the vibrational frequency of the *i^th^* mode. Consequently, the equilibrium isotope fractionation factor between Phases A and B can be expressed as:

${10}^{3}ln\alpha_{A-B}={10}^{3}ln\beta_{A}-{10}^{3}ln\beta_{B}$ (3)

**Theoretical results**

***Ni-bearing structures***: Nickel is a minor element in the major silicate minerals. The Ni mainly substitutes for the octahedral Mg site in the silicate minerals through Mg^2+^↔Ni^2+^. Forsterite, the Mg end-member of olivine (Mg_2_SiO_4_) has two nonequivalent octahedral Mg sites [M1(4a) and M2(4c)] and the M2 site is slightly larger than the M1 site. Because Ni^2+^ has a slightly smaller ionic radius than Mg^2+^, it is expected that Ni^2+^ would preferentially enter the M1 site. Our calculations demonstrate that the incorporation of Ni^2+^ into the M1 site has a much lower total energy than the Ni^2+^ in the M2 site, confirming that Ni^2+^ is mainly accommodated into the M1 site in olivine. This conclusion is also supported by the experimental observation that most of Ni atoms in synthetic Ni-Mg olivine solid solutions are concentrated in the octahedral M1 sites^10^. The orthorhombic Mg end-member of wadsleyite has three types of Mg sites [M1(4a), M2(4e) and M3(8g)]. We also investigated three different configurations that Ni^2+^ is incorporated into the M1, M2, and M3 sites, respectively. The configuration with Ni^2+^ in the M1 site has the lowest total energy, while the energy difference between the configurations with Ni^2+^ in the M1 and M3 site is quite small. The most stable structural configuration was selected to calculate the vibrational properties and the ﻿reduced partition function ratio. Ringwoodite and bridgmanite have equivalent Mg sites and the Ni-bearing can be obtained by replacing Mg atoms with Ni atoms. Similarly, the Fe-Ni substitution in hcp iron can generate the initial structures of hcp Fe-Ni alloy. To evaluate the effect of sulfur (S) on the β factor, we also investigated the hcp Fe_14_NiS alloy by incorporating one S atom into the Fe site. This substitution yields several nonequivalent configurations and the ﻿structure with the lowest total energy was used.

Because the mineral concentration variation may significantly affect the equilibrium isotope fractionation^11,12^, we also investigated the effect of mineral Ni concentration on the equilibrium inter-mineral Ni isotope fractionation factors. The initial structures of olivine, wadsleyite, ringwoodite, bridgmanite, and Fe-Ni alloy with different Ni contents were obtained by incorporating Ni into their supercells, a procedure similar to the Ca-Mg substitutions in our previous studies^13,14^. For instance, the initial structure of olivine with Ni/(Ni+Mg) of 1/32 can be obtained by replacing one M1-site Mg atom in the Mg_32_Si_16_O_64_ supercell with one Ni atom.

***Average Ni-O bond lengths*:** The calculated average Ni-O bond lengths and Ni coordination numbers (CNs) in Ni-doped silicate minerals are listed in Supplementary Table 8. The average Ni-O bond length and Ni CN depend on the accepted threshold of Ni-O bond lengths. Our calculations show that Ni-O distances in all calculated silicate minerals range from 2.0 Å to 2.2 Å or greater than 2.4 Å at ambient pressure, thus we adopted the value of 2.2 Å as the cutoff for Ni-O bond lengths. The Ni CNs in olivine, wadsleyite, ringwoodite, and bridgmanite are all six. In olivine and wadsleyite, the average Ni-O bond lengths are insensitive to their Ni concentration when Ni/(Ni+Mg) in olivine and wadsleyite are lower than 1/32 and 1/16, respectively. In ringwoodite, the average Ni-O bond length decreases significantly from 2.0922 to 2.0694 Å as Ni/(Ni+Mg) decreases from 1/16 to 1/32, but it does not change with Ni content when Ni/(Ni+Mg) is lower than 1/32. Similarly, the average Ni-O bond length in bridgmanite is only sensitive to Ni content when 1/32 < Ni/(Ni+Mg) < 1/16. Because naturally occurred silicate minerals usually have Ni/(Ni+Mg) ratios lower than 1/32, the variation of Ni concentration shows a negligible effect on the average Ni-O bond length.

In addition, the average Ni-O bond length in olivine, wadsleyite, ringwoodite, and bridgmanite decreases significantly with compression, similar to the dependences of Mg-O and Si-O bond lengths on pressure^15^. For instance, the Ni-O bond length in olivine decreases from 2.1069 Å at 0 GPa to 2.0558 Å at 14 GPa. The phase transition from olivine to wadsleyite at 14 GPa and from wadsleyite to ringwoodite at 18 GPa only cause a ~1.0% and ~0.3% decreases in the average Ni-O bond length respectively, which is consistent with the volume sequence of olivine > wadsleyite > ringwoodite. In contrast, bridgmanite has a longer average Ni-O bond length than ringwoodite at 25 GPa, although bridgmanite is denser than ringwoodite.

***The reduced partition function ratios (10^3^lnβ) of ^60^Ni/^58^Ni***: The 10^3^lnβ of ^60^Ni/^58^Ni of all calculated minerals are shown in Supplementary Figure 3 and ﻿their polynomial fitting factors with temperature are listed in Supplementary Table 9. Similar to the dependences of average Ni-O bond length on Ni content, the 10^3^lnβ of olivine and wadsleyite at ambient pressure are insensitive to Ni/(Ni+Mg) ratio within the explored compositional space. The 10^3^lnβ of Fe-Ni alloy also does not significantly change with Ni content when Ni/(Ni+Fe) is lower than 1/16. In contrast, the 10^3^lnβ of ringwoodite and bridgmanite at ambient pressure increase with decreasing Ni content when 1/32 < Ni/(Ni+Mg) < 1/16, while they maintain constant when Ni/(Ni+Mg) is lower than 1/32. At ambient pressure, the 10^3^lnβ of ^60^Ni/^58^Ni decreases in the order of olivine ~ wadsleyite ~ bridgmanite < ringwoodite. However, at 25 GPa, this order is bridgmanite < olivine ~ wadsleyite ~ ringwoodite, because the 10^3^lnβ of those minerals show different increasing dependences on pressure. The olivine-wadsleyite and wadsleyite-ringwoodite phase transitions at 14 and 18 GPa increase the 10^3^lnβ by only ~0.02‰ at 1000 K, while the 10^3^lnβ of bridgmanite at 1000 K is ~0.11‰ lower than that of ringwoodite. In addition, the 10^3^lnβ of hcp Fe-Ni and Fe-Ni-S alloy is comparable to that of bridgmanite.

The relative difference in 10^3^lnβ is controlled by the chemical bond strength. In general, shorter chemical bonds are stronger with higher vibrational frequencies and enriched in heavier isotopes relative to longer and weaker chemical bonds^16-18^. To quantify the dominant factor of 10^3^lnβ, we calculated the force constant of Ni in all minerals from the partial phonon density of state *g(E)* of Ni following the equation Dauphas et al.^19^:

$<F>=\frac{M}{\hbar^{2}}\int_{0}^{+\infty} E^{2}g(E)dE$ (4)

where *M* is the mass of Ni, and $\hbar$ is the reduced Planck constant. As shown in Supplementary Figure 4, the 10^3^lnβ of ^60^Ni/^58^Ni at 2000 K are linearly, positively correlated with *<F>* of Ni. The Ni concentration effect on the 10^3^lnβ of ringwoodite and bridgmanite at a certain concentration range can be well explained by the increasing of <F> with decreasing Ni/(Ni+Mg) ratio. Likewise, the incorporation of S into the Fe-Ni alloy slightly decreases the <F> of Ni and hence mildly decreases the 10^3^lnβ value.

Under high temperature of mantle, the equilibrium Ni isotope fractionation factors (10^3^lnα of ^60^Ni/^58^Ni) among olivine, wadsleyite, ringwoodite, and bridgmanite is negligible. For example, at 2000 K, the 10^3^lnα_wadsleyite-olivine_ at 14 GPa and 10^3^lnα_ringwoodite-wadsleyite_ is only 0.005‰ and 0.007‰ respectively; 10^3^lnα_bridgmanite-ringwoodite_ at 25 GPa is only -0.03‰. This implies that there is no significant difference in Ni isotope composition between the upper mantle and lower mantle during the solidification of the magma ocean. Therefore, the Ni isotope composition of bulk silicate Earth should be identical to that of the upper mantle and is lighter than the chondritic value.

On the other hand, the 10^3^lnα between hcp Fe-Ni alloy and bridgmanite depends on pressure. Compared to bridgmanite, Fe-Ni alloy is mildly enriched in heavy Ni isotopes at 25 GPa but enriched in light Ni isotopes at 40 GPa, because of the larger pressure effect on the <F> of Ni in bridgmanite than that in Fe-Ni alloy. However, because the <F> in bridgmanite shows a nonlinear dependence on pressure at 40-60 GPa, Fe-Ni alloy has a larger <F> than bridgmanite at 60 GPa and is thus relatively enriched in heavy Ni isotopes (Supplementary Tables 7 and 8). The incorporation of S into Fe-Ni alloy decreases the <F> of Ni, especially at 40 GPa, which results in the enrichment of light Ni isotopes in Fe-Ni-S alloy relative to bridgmanite. Overall, the 10^3^lnα between hcp Fe-Ni (S) alloy and bridgmanite under the core-forming conditions is <0.01‰.

**Supplementary Note 3: diffusion model**

The behavior of nickel isotopes during chemical diffusion is simulated between a molten mantle and a solid core. The diffusion in the two phases are modeled using a one-dimensional diffusion model separately, as each side requires different diffusivities.

The interface between the core and mantle is set as x = 0. The initial effective Ni concentration (activity) at x<0 (core) is C_C_, and that in the x>0 half (mantle) is C_M_. The partition coefficient of Ni between the two phases is K (K^core/mantle^). The diffusivity in core half (x<0) is D_C_, and in the mantle half (x>0) is D_M_. The following equations are used:

Diffusion equation: $\frac{\partial C}{D\partial t}=\frac{\partial^{2}C}{\partial x^{2}}$.

Initial condition: $C_{t=0}=\left\{ \begin{aligned} C_{C} x<0 \\ C_{M} x>0 \end{aligned} \right.$.

Using the solution from Zhang ^20^, we get:

$C=C_{C}+\frac{\gamma(C_{M}-KC_{C})}{1+K\gamma}erfc\frac{\left| x \right|}{2\sqrt{D_{C}t}}$, when x<0;

$C=C_{M}+\frac{KC_{C}-C_{M}}{1+K\gamma}erfc\frac{x}{2\sqrt{D_{M}t}}$, when x>0.

Where γ = (ρ_M_/ρ_C_) (D_M_/ D_C_)^1/2^, ρ_M_ and ρ_C_ are densities of the lower mantle (～5.6 g/cm^3^) and the outer core (～10.0 g/cm^3^), respectively; erfc is Gauss error function; *t* is the duration of diffusion. The initial Ni content of core (C_C_) and mantle (C_M_) are set as 5.6 wt. % and 0.2 wt. %, respectively. The diffusivity in mantle (D_M_) is assumed to be 10 times higher than that in core (D_C_). The diffusion is modeled under two different distribution coefficients: K = 10 and K = 25. All the isotopes are treated as individual elements. The Ni isotope value of both initial core and mantle is assumed to be chondritic, δ^60^Ni = 0.23. The ratio of diffusivities of isotopes was expressed as D_2_/D_1_ = (m_1_/m_2_)^β^ ^21^. Based on the experimental study on Fe–Ni alloy system^22^, the β value in the core side (x<0) adopted here is 0.32. Since the β value in the mantle is unknown, it is approximated by using the β value in olivine (β = 0.09)^23^. The diffusivity of Ni in the core is estimated to be 42.17 um^2^ per second at 2500 K based on the fitting function of Ni diffusion in Fe-Ni alloy system^22^. The duration of diffusion is set as 1 Myr, 10 Myr and 100 Myr, respectively. The modelling results show that the scale of Ni isotope fractionation is related to the distribution coefficient of Ni between core and mantle (Supplementary Figure 7). The range of diffusion on a 100 Myr time scale is no farther than 6000 meters in the mantle and no farther than 2000 meters in the core (Supplementary Figure 7).

**Supplementary Figure. 1**.

Ni isotope compositions of a variety of geological reference materials measured in this and previous studies. IU, Indiana University; CUGB, China University of Geosciences (Beijing). Data are reported in Supplementary Table 1.

**Supplementary Figure. 2**

Variations of δ^60^Ni against Mg number (Mg#) and Al_2_O_3_ for all published peridotites. Red squares are peridotite samples from this study, the black cycles are from *Klaver et al.* ^2^ and the black diamonds are from *Gall et al.* ^1^. Data are presented in Supplementary Table 4. The fertile and non-metasomatized peridotites with chemical compositions most close to the Bulk Silicate Earth^3^ have homogeneous Ni isotopic compositions, while those experienced secondary modification have relatively large variations. Therefore, only fertile, non-metasomatized peridotites are taken for the average calculation (0.10 ± 0.07‰), shown as the green cross. Error bars represent 2s.d.

**Supplementary Figure. 3**

Temperature dependences of calculated ﻿reduced partition function ratios (10^3^lnβ) of ^60^Ni/^58^Ni for Ni-doped silicate minerals and Fe-Ni(S) alloy.

**Supplementary Figure. 4**

The relationship between 10^3^lnβ of ^60^Ni/^58^Ni at 2000 K and force constant of Ni in all calculated minerals.

**Supplementary Figure. 5**

Pressures of silicate and Fe-Ni-Si melts predicted by the first-principles molecular dynamic simulations at 3000 K. The pressure is 37.6 GPa for Mg_30_Ni_2_Si_32_O_96_ melt and 38.5 GPa for Fe_92_Ni_5_S_3_ melt.

**Supplementary Figure. 6**

Force constants of Ni in captured snapshots (grey scatters) from Mg_30_Ni_2_Si_32_O_96_ and Fe_92_Ni_5_S_3_ melts and their cumulative averages (red and blue scatters) in the time domain.

**Supplementary Figure. 7**

Modeled Ni isotopic diffusion profile at the core-mantle boundary. Detail information on the modeling parameters and interpretation are given in the Supplementary Note 3.

**Supplementary Figure. 8**

Mass balance model estimates the influence of late-stage impactors on the Ni isotope composition of the proto-BSE. The δ^60^Ni value of the proto-BSE is assumed to be chondrite-like, that is +0.23‰. The δ^60^Ni value of the impactor is assumed to be -2.5‰, -1.0‰, -0.5‰, and +0.1‰, respectively. Accretion models imply that the Moon-forming impactor contributed ~20% (*f*) of Ni of the BSE (black triangle) and the late veneer materials added less than 5% (*f*)^24^. The mass balance calculation suggests that to account for the observed sub-chondritic Ni isotope composition of the BSE (grey band), the late veneer is required to have extremely low δ^60^Ni of ~ -2.5‰, which is inconsistent with the isotopic signature of the carbonaceous chondrite-like nature for the late veneer inferred from recent studies^25-28^. Therefore, the sub-chondritic Ni isotope signature of the BSE was likely delivered by the Moon-forming impactor with δ^60^Ni values of ~ -0.35‰.

Supplementary Table 1.

Ni isotope compositions of rock standards.

| Standard | δ^60^Ni | 2SD | Reference |
| --- | --- | --- | --- |
| BHVO-1 | 0.05 | 0.05 | 1 |
|  | 0.08 | 0.03 | 2 |
|  | 0.03 | 0.03 | 3 |
| BHVO-2 | 0.04 | 0.01 | 1* |
|  | 0.08 | 0.02 | 2 |
|  | 0.01 | 0.04 | 4 |
|  | 0.13 | 0.03 | 5 |
|  | 0.06 | 0.05 | 6 |
|  | -0.01 | 0.05 | 7 |
| BIR-1 | 0.17 | 0.02 | 1 |
|  | 0.19 | 0.07 | 2 |
|  | 0.12 | 0.03 | 3 |
|  | 0.12 | 0.04 | 4 |
| DTS-1 | -0.08 | 0.03 | 1 |
|  | -0.07 | 0.01 | 2 |
|  | -0.07 | 0.05 | 4 |
|  | -0.08 | 0.07 | 8 |
|  | -0.08 | 0.03 | 9 |
| JP-1 | 0.13 | 0.01 | 1* |
|  | 0.13 | 0.03 | 2 |
|  | 0.12 | 0.01 | 9 |
|  | 0.10 | 0.08 | 10 |
| BCR-2 | 0.23 | 0.07 | 1 |
|  | 0.18 | 0.01 | 1* |
|  | 0.20 | 0.07 | 5 |
|  | 0.20 | 0.08 | 6 |
| Nod-A-1 | 1.08 | 0.06 | 1 |
|  | 1.10 | 0.07 | 3 |
|  | 1.03 | 0.06 | 4 |
|  | 1.08 | 0.06 | 11 |
|  | 1.11 | 0.03 | 12 |
|  | 1.10 | 0.10 | 13 |
|  | 1.08 | 0.09 | 14 |
| Nod-P-1 | 0.33 | 0.08 | 1 |
|  | 0.36 | 0.07 | 4 |
|  | 0.41 | 0.06 | 11 |
|  | 0.38 | 0.03 | 12 |
|  | 0.41 |  | 13 |
|  | 0.36 | 0.07 | 14 |
| SCO-1 | 0.11 | 0.04 | 1 |
|  | 0.08 | 0.07 | 3 |
|  | 0.08 | 0.08 | 14 |
| SDO-1 | 0.60 | 0.05 | 3 |
|  | 0.59 | 0.03 | 4 |
|  | 0.54 | 0.05 | 7 |
|  | 0.57 | 0.09 | 14 |

Standards labeled with * are analyzed at CUGB, while others are analyzed at IU. 1, this study; 2, Chemonzhkin et al., ^29^; 3, Wang et al.,^5^; 4, Gueguen et al., ^7^; 5, Cameron et al., ^6^; 6, Gall et al., ^30^; 7, Estrade et al., ^31^; 8, Gall et al., ^1^; 9, Klaver et al., ^2^; 10, Steele et al., ^32^; 11, Gall et al., ^33^; 12, Ratie et al., ^34^; 13, Cameron and Vance, ^35^; 14, Wang and Wasylenki, ^36^.

Supplementary Table 2.

Mass-independent and -dependent Ni isotope compositions in chondrites and iron meteorites.

| **Sample** | **Class** | **δ^60^Ni** | **2SD** | **ε^62^Ni^a^** | **2sd** | **Reference** |
| --- | --- | --- | --- | --- | --- | --- |
| Orgueil | CI | 0.21 | 0.07 |  |  | 2 |
| Orgueil |  | 0.19 | 0.05 |  |  | 3 |
| Orgueil |  | 0.18 | 0.04 |  |  | 4 |
| Orgueil |  | 0.02 | 0.01 |  |  | 5 |
| Orgueil |  | 0.12 | 0.01 |  |  | 5 |
| Ivuna |  | 0.11 | 0.01 |  |  | 5 |
|  | **Ave. CI** | **0.13** | **0.04** | **0.20** | **0.11** |  |
| Murchison | CM | 0.21 | 0.03 |  |  | 2 |
| Murchison |  | 0.23 | 0.07 |  |  | 4 |
| Murchison |  | 0.19 | 0.01 |  |  | 5 |
|  | **Ave. CM** | **0.21** | **0.04** | **0.10** | **0.05** |  |
| Felix | CO | 0.31 | 0.07 |  |  | 2 |
| Ornans |  | 0.29 | 0.08 |  |  | 4 |
| Omans |  | 0.21 | 0.01 |  |  | 5 |
| Kainsaz |  | 0.20 | 0.01 |  |  | 5 |
|  | **Ave. CO** | **0.25** | **0.11** | **0.11** | **0.04** |  |
| Renazzo | CR | 0.16 | 0.01 |  |  | 5 |
| Al Rais |  | 0.22 | 0.01 |  |  | 5 |
|  | **Ave. CR** | **0.19** | **0.08** | **0.09** | **0.06** |  |
| Leoville | CV | 0.30 | 0.05 |  |  | 2 |
| Allende |  | 0.24 | 0.07 |  |  | 4 |
| Allende |  | 0.243 | 0.01 |  |  | 5 |
| Allende |  | 0.25 | 0.04 |  |  | 1 |
| Kaba |  | 0.22 | 0.04 |  |  | 1 |
|  | **Ave. CV** | **0.26** | **0.08** | **0.11** | **0.07** |  |
| Karoonda | CK | 0.278 | 0.008 |  |  | 5 |
| Karoonda |  | 0.39 | 0.04 |  |  | 1 |
|  | **Ave. CK** | **0.33** | **0.16** |  |  |  |
| Abee | EH | 0.19 | 0.05 |  |  | 2 |
| Abee |  | 0.25 | 0.01 |  |  | 5 |
| Indarch |  | 0.27 | 0.09 |  |  | 4 |
| Indarch |  | 0.19 | 0.01 |  |  | 5 |
| St.Marks |  | 0.18 | 0.01 |  |  | 5 |
| Kota-kota |  | 0.20 | 0.01 |  |  | 5 |
| Kota-kota |  | 0.26 | 0.04 |  |  | 1 |
| Kota-kota |  | 0.21 | 0.04 |  |  | 1 |
|  | **Ave. EH** | **0.21** | **0.04** | **0.03** | **0.04** |  |
| Khairpur | EL | 0.29 | 0.08 |  |  | 4 |
| Khairpur |  | 0.21 | 0.01 |  |  | 5 |
| Atlanata |  | 0.21 | 0.01 |  |  | 5 |
| Hvittis |  | 0.22 | 0.01 |  |  | 5 |
| Yimia |  | 0.21 | 0.01 |  |  | 5 |
|  | **Ave. EL** | **0.22** | **0.04** | **-0.03** | **0.09** |  |
| A-10224 (L3), bulk | L | 0.24 | 0.01 |  |  | 6 |
| Barratta |  | 0.31 | 0.05 |  |  | 4 |
| Barratta |  | 0.19 | 0.01 |  |  | 5 |
| Bruderheim |  | 0.51 | 0.07 |  |  | 4 |
| Ceniceros |  | 0.20 | 0.01 |  |  | 5 |
|  | **Ave. L^b^** | **0.23** | **0.05** | **-0.04** | **0.04** |  |
| Chainpur | LL | 0.28 | 0.10 |  |  | 2 |
| Chainpur |  | 0.28 | 0.04 |  |  | 1 |
| A-09135 (LL3), bulk |  | 0.15 | 0.02 |  |  | 6 |
| Parnallee |  | 0.16 | 0.05 |  |  | 4 |
| Parnallee |  | 0.20 | 0.01 |  |  | 5 |
| St Severin |  | 0.24 | 0.05 |  |  | 4 |
| Chelyabinsk |  | 0.27 | 0.01 |  |  | 5 |
| Dhurmsala |  | 0.22 | 0.01 |  |  | 5 |
| Kilabo |  | 0.25 | 0.01 |  |  | 5 |
|  | **Ave. LL** | **0.23** | **0.09** | **-0.07** | **0.05** |  |
| A-09436 (H3), bulk | H | 0.20 | 0.04 |  |  | 6 |
| Bremervorde |  | 0.26 | 0.05 |  |  | 4 |
| Kernouve |  | 0.37 | 0.08 |  |  | 4 |
| Buzzard Coulee |  | 0.17 | 0.01 |  |  | 5 |
|  | **Ave. H** | **0.25** | **0.18** | **-0.06** | **0.08** |  |
| Darinskoe | IIC | **0.23** | **0.08** | **0.16** | **0.09** | 2 |
| Nantan iron | IID | **0.32** | **0.06** | **0.19** | **0.06** | 7 |
| BM 1959.949 | IIIF | **0.30** | **0.05** | **0.12** | **0.08** | 4 |
| Chinga bulk | IVB | 0.22 | 0.03 |  |  | 6 |
| Hoba |  | 0.33 | 0.06 |  |  | 2 |
| Santa Clara |  | 0.32 | 0.11 |  |  | 8 |
|  | **Ave. IVB** | **0.29** | **0.12** | **0.08** | **0.03** |  |
| USNM 2638 | IC | **0.20** | **0.06** | **-0.05** | **0.07** | 4 |
| Sikhote Alin | IIAB | 0.23 | 0.11 |  |  | 6 |
| Coahuila |  | 0.36 | 0.04 |  |  | 2 |
|  | **Ave. IIAB** | **0.30** | **0.18** | **-0.07** | **0.04** |  |
| Henbury | IIIAB | 0.24 | 0.07 | **-0.11** | **0.07** | 2 |
| Duel Hill | IVA | -0.06 | 0.06 |  |  | 4 |
| Gibeon |  | 0.31 | 0.06 |  |  | 4 |
| Bristol |  | 0.28 | 0.05 |  |  | 2 |
| Gibeon |  | 0.26 | 0.05 |  |  | 7 |
|  | **Ave. IVA^b^** | **0.28** | **0.05** | **-0.07** | **0.10** |  |

^a^, Nucleosynthetic Ni isotope data are from *Refs* ^32,37-42^; ^b^, averages are made by excluding one outlier.

1, this study; 2, Cameron et al., ^6^; 3, Steele et al.,^40^; 4, Gall et al., ^1^; 5, Klaver et al., ^2^; 6, Chemonzhkin et al., ^43^, 7, Gueguen et al., ^7^; 8, Steele et al., ^32^.

Supplementary Table 3.

Sources of the petrology, mineralogy, and elemental composition of the terrestrial samples

| Terrestrial samples | Sources |
| --- | --- |
| Kuandian Peridotites | *Ref.* ^44^ |
| Fansi Peridotites | *Ref.* ^45^ |
| Damaing Peridotites | *Ref.* ^46^ |
| Yangyuan Peridotites | *Refs.* ^45,47,48^ |
| Turon de Tecouere Peridotites | *Ref. ^49^* |
| Komatiites | *Refs*. ^50,51^ |
| Oceanic basalts | *Refs*. ^52,53^ |
| Eclogites | *Refs*. ^54,55^ |

Supplementary Table 4.

Ni isotope compositions of peridotites.

| **Sample** | **Location** | **Rock type** | **Metasomatism** | **δ^60^Ni** | **2SD** | **Mg#** | **Al_2_O_3_** | **Ni** |
| --- | --- | --- | --- | --- | --- | --- | --- | --- |
|  |  |  |  | **‰** |  |  | ***wt*%** | **ppm** |
| ***This study*** |  |  |  |  |  |  |  |  |
| HY1-01 | Kuandian | harzburgite | yes | 0.12 | 0.06 | 90.4 | 1.6 | 1907 |
| HY2-01 | Kuandian | lherzolite | yes | 0.14 | 0.06 | 89.6 | 2.2 | 1821 |
| HY2-02 | Kuandian | lherzolite | no | 0.09 | 0.03 | 88.6 | 3.1 | 1332 |
| HY2-03 | Kuandian | lherzolite | no | 0.13 | 0.04 | 89.1 | 3.1 | 1830 |
| HY2-04 | Kuandian | lherzolite | no | 0.07 | 0.08 | 88.4 | 2.6 | 1789 |
| HY2-05 | Kuandian | harzburgite | yes | 0.11 | 0.06 | 90.4 | 1.2 | 1952 |
| HY2-06 | Kuandian | lherzolite | yes | -0.08 | 0.05 | 89.2 | 2.6 | 1783 |
| HY2-07 | Kuandian | lherzolite | no | 0.11 | 0.09 | 88.3 | 4.1 | 1237 |
| HY2-14 | Kuandian | lherzolite | no | 0.04 | 0.07 | 89.7 | 2.6 | 1825 |
| HY2-29 | Kuandian | lherzolite | no | 0.09 | 0.04 | 88.3 | 4.3 | 1663 |
| FS-01* | Fansi | lherzolite | yes | 0.07 | 0.03 | 89.6 | 1.9 | 2366 |
| FS-04* | Fansi |  | yes | 0.21 | 0.04 | 91.5 | 0.9 | 2636 |
| Replicate |  |  |  | 0.18 | 0.03 |  |  |  |
| Replicate |  |  |  | 0.20 | 0.03 |  |  |  |
| Average. |  |  |  | 0.20 | 0.02 |  |  |  |
| FS-18* | Fansi | harzburgite | yes | 0.16 | 0.03 | 91.0 | 2.0 | 2071 |
| Replicate |  |  |  | 0.09 | 0.03 |  |  |  |
| Average. |  |  |  | 0.12 | 0.10 |  |  |  |
| FS-36* | Fansi | lherzolite | yes | 0.07 | 0.03 | 89.2 | 3.5 | 2176 |
| FS-44* | Fansi | lherzolite | yes | 0.05 | 0.03 | 90.0 | 2.2 | 2127 |
| FS-45* | Fansi | lherzolite | yes | 0.10 | 0.03 | 89.5 | 2.5 | 1957 |
| FS-50* | Fansi | lherzolite | yes | 0.13 | 0.04 | 88.7 | 2.6 | 1926 |
| FS-64* | Fansi |  | yes | 0.17 | 0.03 | 89.3 | 2.0 | 2394 |
| FS2-04* | Fansi | harzburgite | yes | 0.16 | 0.04 | 90.4 | 1.5 | 2085 |
| DMP-04* | Damaping | lherzolite | no | 0.05 | 0.03 | 91.1 | 2.3 | 2376 |
| DMP-60* | Damaping | lherzolite | yes | 0.10 | 0.03 | 89.7 | 3.7 | 2148 |
| YY-22* | Yangyuan | lherzolite | yes | 0.01 | 0.03 | 91.0 | 2.5 | 1995 |
| YY-27* | Yangyuan | lherzolite | yes | 0.01 | 0.03 | 89.6 | 2.9 | 2047 |
| YY-51* | Yangyuan | harzburgite | yes | -0.05 | 0.04 | 91.4 | 1.1 | 2500 |
| Replicate |  |  |  | -0.08 | 0.01 |  |  |  |
| Average. |  |  |  | -0.06 | 0.04 |  |  |  |
| YY-52* | Yangyuan | lherzolite | yes | 0.15 | 0.04 | 90.6 | 2.7 | 2115 |
| TT16* | Turon de Tecouere | lherzolite | no | 0.08 | 0.09 | 89.5 | 3.9 | 1948 |
| TT24* | Turon de Tecouere | lherzolite | no | 0.14 | 0.07 | 88.9 | 4.2 | 1826 |
| TT27* | Turon de Tecouere | lherzolite | no | 0.12 | 0.04 | 88.7 | 4.2 | 1787 |
| TT02* | Turon de Tecouere | lherzolite | no | 0.10 | 0.04 | 88.9 | 3.9 | 2115 |
| TT11* | Turon de Tecouere | lherzolite | no | 0.12 | 0.04 | 89.3 | 3.8 | 2106 |
| TT12* | Turon de Tecouere | lherzolite | no | 0.11 | 0.08 | 89.4 | 3.7 | 2109 |
| TT13* | Turon de Tecouere | lherzolite | no | 0.14 | 0.09 | 89.3 | 3.7 | 2152 |
| TT21* | Turon de Tecouere | lherzolite | no | 0.15 | 0.09 | 89.1 | 4.0 | 2037 |
| ***Klaver et al., 2019*** |  |  |  |  |  |  |  |  |
| BZ116 | Horoman | harzburgite | no | 0.11 |  | 91.38 | 0.5 | 2367 |
| BZ117 | Horoman | harzburgite | no | 0.15 |  | 91.42 | 0.8 | 2327 |
| BZ131 | Horoman | harzburgite | no | 0.12 |  | 91.28 | 0.6 |  |
| BZ216 | Horoman | harzburgite | no | 0.11 |  | 91.50 | 0.8 | 2375 |
| BZ203 | Horoman | lherzolite | no | 0.11 |  | 90.90 | 1.3 | 2320 |
| BZ134 | Horoman | lherzolite | no | 0.13 |  | 91.28 | 1.4 | 2336 |
| BZ143 | Horoman | lherzolite | no | 0.07 |  | 90.53 | 2.0 | 2140 |
| BZ251 | Horoman | lherzolite | no | 0.08 |  | 89.90 | 3.2 | 2001 |
| BZ252 | Horoman | lherzolite | no | 0.12 |  | 89.52 | 3.5 | 1996 |
| 312-102 | Vitim | lherzolite | no | 0.13 |  | 89.00 | 4.8 | 1283 |
| 313-105 | Vitim | lherzolite | no | 0.15 |  | 89.86 | 3.2 | 2059 |
| 313-240 | Vitim | lherzolite | no | 0.13 |  | 89.62 | 3.4 | 2051 |
| 314-56 | Vitim | lherzolite | no | 0.10 |  | 88.77 | 4.1 | 1886 |
| 314-58 | Vitim | lherzolite | no | 0.10 |  | 89.31 | 3.8 | 1965 |
| BZ-26 | Zabargad | lherzolite | no | 0.13 |  | 90.25 | 2.1 | 2289 |
| BZ-29 | Zabargad | lherzolite | no | 0.13 |  | 88.80 | 3.8 | 1983 |
| BZ-230 | Zabargad | lherzolite | yes | 0.22 |  | 88.93 | 3.7 | 2007 |
| BZ-241 | Zabargad | harzburgite | no | 0.04 |  | 90.26 | 1.4 | 2369 |
| S-1 | Tariat | lherzolite | no | 0.12 |  | 89.13 | 4.3 | 1891 |
| S-2 | Tariat | lherzolite | no | 0.12 |  | 89.40 | 3.9 | 2103 |
| S-16 | Tariat | lherzolite | yes | 0.11 |  | 90.30 | 3.0 | 2003 |
| s-62 | Tariat | harzburgite | yes | 0.15 |  | 91.28 | 1.7 | 2113 |
| H-25 | Tariat | harzburgite | yes | 0.15 |  | 91.75 | 0.6 | 2712 |
| 4230/16 | Tariat | lherzolite | yes | 0.16 |  | 88.69 | 4.3 | 1807 |
| ***Gall et al., 2017*** |  |  |  |  |  |  |  |  |
| NHM-2 | South Africa | lherzolite | yes | 0.22 |  | 91.22 | 2.1 | 1740 |
| BD744 | Lashaine | pyroxenite | yes | 0.36 |  | 92.0 |  | 2700 |
| BD822 | Lashaine | harzburgite | yes | 0.18 |  | 92.55 | 1.9 | 2345 |
| BD1542 | Lashaine | harzburgite | yes | 0.20 |  | 92.36 | 0.7 | 2500 |
| BD774 | Lashaine | harzburgite | yes | 0.28 |  | 92.27 | 0.2 | 2165 |
| BD730 | Lashaine | lherzolite | yes | 0.23 |  | 91.27 | 2.4 | 1880 |
| BD1355 | Matsoka | lherzolite | yes | 0.25 |  | 93.06 | 1.5 | 2100 |

Samples labeled with * are analyzed at CUGB, while others are analyzed at IU. Literature data for peridotites are from *Refs* ^1,2^.

Supplementary Table 5.

Ni isotope compositon of komatiites and oceanic basalts

| **Sample** | **Rock type** | **δ^60^Ni** | **2SD** | **Ni** |
| --- | --- | --- | --- | --- |
|  |  | **‰** |  | **ppm** |
| ***Komatiite*** |  |  |  |  |
| A-2 | Alexo, Ontario | 0.14 | 0.06 | 1158 |
| m-1 | Munro Township, Ontario | 0.12 | 0.06 | 937 |
| P-2 | Thompson Manitoba | 0.16 | 0.05 | 2520 |
| Kal-1 | Alexo, Ontario | 0.17 | 0.06 | 1332 |
| SC5^a^ | Komati, Barberton | 0.10 | 0.10 | 2696 |
| SC7^a^ | Komati, Barberton | 0.16 | 0.02 | 847 |
| SC9^a^ | Komati, Barberton | 0.11 | 0.07 | 1947 |
| SC15^a^ | Komati, Barberton | 0.14 | 0.05 | 888 |
| HG5^a^ | Hooggenoeg, Barberton | 0.18 | 0.05 | 1156 |
| HG15^a^ | Hooggenoeg, Barberton | 0.11 | 0.05 | 1037 |
| HG16^a^ | Hooggenoeg, Barberton | 0.07 | 0.07 | 1136 |
| SS1^a^ | Sandspruit, Barberton | 0.02 | 0.04 | 1690 |
| SS5^a^ | Sandspruit, Barberton | 0.09 | 0.06 | 1594 |
| SS6^a^ | Sandspruit, Barberton | 0.13 | 0.04 | 1181 |
| SS8^a^ | Sandspruit, Barberton | 0.20 | 0.08 | 1512 |
| GOR94-3^b^ | Gorgona | 0.22 | 0.04 | 1575 |
| GOR94-17^b^ | Gorgona | 0.15 | 0.05 | 1110 |
| GOR94-44^b^ | Gorgona | 0.19 | 0.04 | 1325 |
| GOR94-19^b^ | Gorgona | 0.26 | 0.07 | 1080 |
| GOR94-43^b^ | Gorgona | 0.24 | 0.06 | 1200 |
| ***Oceanic Island Basalt (OIB)*** |  |  |  |  |
| KI-75-1-121.5 | Kilauea Iki lava | -0.04 | 0.08 | 169 |
| Replicate |  | -0.04 | 0.04 |  |
| Average |  | -0.04 | 0.00 |  |
| IKI-58 | Kilauea Iki lava | 0.08 | 0.02 | 133 |
| KI-67-3-39 | Kilauea Iki lava | 0.04 | 0.09 | 340 |
| KI-75-1-39 | Kilauea Iki lava | 0.06 | 0.07 | 378 |
| KI-79-3-150.4 | Kilauea Iki lava | -0.04 | 0.02 | 520 |
| KI-67-3-80.7 | Kilauea Iki lava | -0.06 | 0.04 | 211 |
| Loihi^c^ | Hawaii | 0.07 | 0.03 | 603 |
| ***Middle Ocean Ridge Basalt (MORB)*** |  |  |  |  |
| 3369-1129 | Hess Deep Rift | 0.03 | 0.03 | 63 |
| 3379-1031 | Hess Deep Rift | 0.21 | 0.04 | 59 |
| 142-864A-001 | Hole 864 East Pacific Rise | 0.01 | 0.08 | 129 |
| CR01-1* | Carlsberg Ridge | -0.01 | 0.01 | 146 |
| CR02-1* | Carlsberg Ridge | -0.00 | 0.02 | 118 |
| CR03-3* | Carlsberg Ridge | 0.17 | 0.01 | 190 |
| CR04-1* | Carlsberg Ridge | 0.01 | 0.01 | 153 |
| Replicate |  | -0.02 | 0.02 |  |
| Average |  | -0.01 | 0.04 |  |
| AR04* | North Atlantic | 0.01 | 0.01 | 144 |
| AR05* | North Atlantic | -0.06 | 0.02 | 151 |
| 2351-2^d^ | East Pacific Rise | 0.13 | 0.06 | 103 |
| 2359-4^d^ | East Pacific Rise | 0.01 | 0.07 | 80 |
| 2372-1^d^ | East Pacific Rise | 0.24 | 0.09 | 110 |
| 1149B-32R1, 136^c^ | IODP site 1149, Hole C | 0.02 | 0.03 | 73 |
| 1149B-29R3, 60/D^c^ | IODP site 1149, Hole C | 0.00 | 0.04 | 71 |
| 1149D-19R1, 85^c^ | IODP site 1149, Hole C | 0.05 | 0.02 | 43 |
| 1149D-06R2, 82^c^ | IODP site 1149, Hole C | -0.03 | 0.03 | 61 |
| 1149D-16R3, 0^c^ | IODP site 1149, Hole C | 0.10 | 0.03 | 111 |

Samples labeled with * are analyzed at CUGB, while others are analyzed at IU. Literature data: a, Wang et al. ^5^; b, Gall et al. ^1^; c, Gueguen et al. ^7^; d, Cameron et al. ^6^

Supplementary Table 6.

Ni isotope compositon of eclogites

| **Sample** | **Location** | **δ^60^Ni** | **2SD** | **Ni** |
| --- | --- | --- | --- | --- |
|  |  | **‰** |  | **ppm** |
| HA-3 | Hongan, Dabie | 0.01 | 0.05 | 153 |
| HA-4 | Hongan, Dabie | 0.01 | 0.03 | 29 |
| SH-1 | Shuanghe, Dabie | 0.07 | 0.04 | 73 |
| 8--0 | Western Tianshan | 0.06 | 0.09 | 45 |
| 8--20 | Western Tianshan | 0.00 | 0.07 | 46 |
| 305-2 | Western Tianshan | 0.04 | 0.03 | 146 |
| H902-7 | Western Tianshan | -0.01 | 0.07 | 53 |

Supplementary Table 7.

The k-point mesh used to calculate Brillouin zone summations over electronic states.

| **Minerals** | **Chemical composition** | **Ni content** | **k-point mesh** |
| --- | --- | --- | --- |
| Olivine | Mg_31_NiSi_16_O_64_ | Ni/(Ni+Mg)=1/32 | 1×1×1 |
|  | Mg_63_NiSi_32_O_128_ | Ni/(Ni+Mg)=1/64 | 1×1×1 |
| Wadsleyite | Mg_15_NiSi_8_O_32_ | Ni/(Ni+Mg)=1/16 | 2×1×2 |
|  | Mg_31_NiSi_16_O_64_ | Ni/(Ni+Mg)=1/32 | 1×1×2 |
|  | Mg_63_NiSi_32_O_128_ | Ni/(Ni+Mg)=1/64 | 1×1×1 |
| Ringwoodite | Mg_15_NiSi_8_O_32_ | Ni/(Ni+Mg)=1/16 | 2×2×2 |
|  | Mg_31_NiSi_16_O_64_ | Ni/(Ni+Mg)=1/32 | 2×2×2 |
|  | Mg_63_NiSi_32_O_128_ | Ni/(Ni+Mg)=1/64 | 1×1×1 |
| Bridgmanite | Mg_15_NiSi_16_O_48_ | Ni/(Ni+Mg)=1/16 | 1×1×2 |
|  | Mg_31_NiSi_32_O_96_ | Ni/(Ni+Mg)=1/32 | 1×1×1 |
|  | Mg_47_NiSi_48_O_144_ | Ni/(Ni+Mg)=1/48 | 1×1×1 |
| Hcp FeNi | Fe_15_Ni | Ni/(Ni+Fe)=1/16 | 8×8×6 |
|  | Fe_63_Ni | Ni/(Ni+Fe)=1/32 | 4×4×4 |
| Hcp FeNiS | Fe_14_NiS | Ni/(Ni+Fe+S)=1/16 | 8×8×6 |

Supplementary Table 8.

Average Ni-O bond lengths, coordination numbers (CN), and force constant of Ni in silicate minerals and Fe-Ni (S) alloy.

| **Minerals** | **Ni content** | **Pressure (GPa)** | **Average Ni-O bond length (Å)** | **CN** | **Force constant <F>** |
| --- | --- | --- | --- | --- | --- |
| Olivine | Ni/(Ni+Mg)=1/32 | 0 | 2.1069 | 6 | 193.9 |
|  | Ni/(Ni+Mg)=1/32 | 14 | 2.0558 | 6 | 245.4 |
|  | Ni/(Ni+Mg)=1/32 | 25 | 2.0167 | 6 | 292.3 |
|  | Ni/(Ni+Mg)=1/64 | 0 | 2.0910 | 6 | 196.4 |
| Wadsleyite | Ni/(Ni+Mg)=1/16 | 0 | 2.0938 | 6 | 194.3 |
|  | Ni/(Ni+Mg)=1/32 | 0 | 2.0954 | 6 | 194.3 |
|  | Ni/(Ni+Mg)=1/32 | 14 | 2.0378 | 6 | 253.8 |
|  | Ni/(Ni+Mg)=1/32 | 18 | 2.0250 | 6 | 269.5 |
|  | Ni/(Ni+Mg)=1/32 | 25 | 2.0051 | 6 | 296.2 |
|  | Ni/(Ni+Mg)=1/64 | 0 | 2.0950 | 6 | 193.5 |
| Ringwoodite | Ni/(Ni+Mg)=1/16 | 0 | 2.0922 | 6 | 194.9 |
|  | Ni/(Ni+Mg)=1/32 | 0 | 2.0694 | 6 | 220.2 |
|  | Ni/(Ni+Mg)=1/32 | 14 | 2.0296 | 6 | 226.0 |
|  | Ni/(Ni+Mg)=1/32 | 18 | 2.0187 | 6 | 280.2 |
|  | Ni/(Ni+Mg)=1/32 | 25 | 2.0027 | 6 | 302.6 |
|  | Ni/(Ni+Mg)=1/64 | 0 | 2.0690 | 6 | 220.7 |
| Bridgmanite | Ni/(Ni+Mg)=1/16 | 0 | 2.1810 | 6 | 168.3 |
|  | Ni/(Ni+Mg)=1/32 | 0 | 2.1060 | 6 | 199.2 |
|  | Ni/(Ni+Mg)=1/32 | 25 | 2.0560 | 6 | 256.0 |
|  | Ni/(Ni+Mg)=1/32 | 40 | 2.0300 | 6 | 310.5 |
|  | Ni/(Ni+Mg)=1/32 | 60 | 2.0017 | 6 | 332.8 |
|  | Ni/(Ni+Mg)=1/32 | 130 | 1.9505 | 6 | 438.8 |
|  | Ni/(Ni+Mg)=1/48 | 0 | 2.1048 | 6 | 200.2 |
| Hcp FeNi | Ni/(Ni+Fe)=1/16 | 25 | - |  | 260.7 |
|  | Ni/(Ni+Fe)=1/16 | 40 | - |  | 298.1 |
|  | Ni/(Ni+Fe)=1/16 | 60 | - |  | 343.9 |
|  | Ni/(Ni+Fe)=1/16 | 130 | - |  | 492.9 |
|  | Ni/(Ni+Fe)=1/64 | 40 | - |  | 293.3 |
|  | Ni/(Ni+Fe)=1/64 | 60 | - |  | 338.2 |
|  | Ni/(Ni+Fe)=1/64 | 130 | - |  | 484.6 |
| Hcp FeNiS | Ni/(Ni+Fe+S)=1/16 | 40 | - |  | 267.5 |
|  | Ni/(Ni+Fe+S)=1/16 | 60 | - |  | 315.3 |

Supplementary Table 9.

Polynomial fitting parameters of the ﻿reduced partition function ratios (10^3^lnβ) of ^60^Ni/^58^Ni for Ni-doped silicate minerals and Fe-Ni (S) alloy.

| **Minerals** | **Ni content** | **Pressure (GPa)** | **a** | **b** | **c** |
| --- | --- | --- | --- | --- | --- |
| Olivine | Ni/(Ni+Mg)=1/32 | 0 | 0.48885 | -2.397E-03 | 2.856E-05 |
|  | Ni/(Ni+Mg)=1/32 | 14 | 0.61868 | -3.034E-03 | 3.614E-05 |
|  | Ni/(Ni+Mg)=1/32 | 25 | 0.73693 | -3.614E-03 | 4.305E-05 |
|  | Ni/(Ni+Mg)=1/64 | 0 | 0.48733 | -2.390E-03 | 2.847E-05 |
| Wadsleyite | Ni/(Ni+Mg)=1/16 | 0 | 0.49031 | -1.911E-03 | 2.002E-05 |
|  | Ni/(Ni+Mg)=1/32 | 0 | 0.48959 | -1.909E-03 | 2.021E-05 |
|  | Ni/(Ni+Mg)=1/32 | 14 | 0.63960 | -3.124E-03 | 3.574E-05 |
|  | Ni/(Ni+Mg)=1/32 | 18 | 0.67926 | -3.529E-03 | 4.176E-05 |
|  | Ni/(Ni+Mg)=1/32 | 25 | 0.74667 | -4.290E-03 | 5.406E-05 |
|  | Ni/(Ni+Mg)=1/64 | 0 | 0.50358 | -2.714E-03 | 3.610E-05 |
| Ringwoodite | Ni/(Ni+Mg)=1/16 | 0 | 0.49137 | -2.410E-03 | 2.871E-05 |
|  | Ni/(Ni+Mg)=1/32 | 0 | 0.55152 | -2.722E-03 | 3.243E-05 |
|  | Ni/(Ni+Mg)=1/32 | 14 | 0.67062 | -3.289E-03 | 3.918E-05 |
|  | Ni/(Ni+Mg)=1/32 | 18 | 0.70642 | -3.464E-03 | 4.127E-05 |
|  | Ni/(Ni+Mg)=1/32 | 25 | 0.76289 | -3.741E-03 | 4.457E-05 |
|  | Ni/(Ni+Mg)=1/64 | 0 | 0.55641 | -2.729E-03 | 3.251E-05 |
| Bridgmanite | Ni/(Ni+Mg)=1/16 | 0 | 0.42431 | -2.081E-03 | 2.479E-05 |
|  | Ni/(Ni+Mg)=1/32 | 0 | 0.50221 | -2.463E-03 | 2.934E-05 |
|  | Ni/(Ni+Mg)=1/32 | 25 | 0.64541 | -3.165E-03 | 3.771E-05 |
|  | Ni/(Ni+Mg)=1/32 | 40 | 0.78281 | -3.839E-03 | 4.573E-05 |
|  | Ni/(Ni+Mg)=1/32 | 60 | 0.83903 | -4.115E-03 | 4.902E-05 |
|  | Ni/(Ni+Mg)=1/32 | 130 | 1.10627 | -5.425E-03 | 6.463E-05 |
|  | Ni/(Ni+Mg)=1/48 | 0 | 0.50473 | -2.475E-03 | 2.949E-05 |
| Hcp FeNi | Ni/(Ni+Fe)=1/16 | 25 | 0.65726 | -3.223E-03 | 3.840E-05 |
|  | Ni/(Ni+Fe)=1/16 | 40 | 0.75155 | -3.686E-03 | 4.391E-05 |
|  | Ni/(Ni+Fe)=1/16 | 60 | 0.86702 | -4.252E-03 | 5.065E-05 |
|  | Ni/(Ni+Fe)=1/16 | 130 | 1.19400 | -5.855E-03 | 6.975E-05 |
|  | Ni/(Ni+Fe)=1/64 | 40 | 0.73945 | -3.626E-03 | 4.320E-05 |
|  | Ni/(Ni+Fe)=1/64 | 60 | 0.85264 | -4.181E-03 | 4.981E-05 |
|  | Ni/(Ni+Fe)=1/64 | 130 | 1.17390 | -5.757E-03 | 6.858E-05 |
| Hcp FeNiS | Ni/(Ni+Fe+S)=1/16 | 40 | 0.67440 | -3.307E-03 | 3.940E-05 |
|  | Ni/(Ni+Fe+S)=1/16 | 60 | 0.79491 | -3.898E-03 | 4.644E-05 |

Supplementary References

1 Gall, L., Williams, H. M., Halliday, A. N. & Kerr, A. C. Nickel isotopic composition of the mantle. *Geochimica et Cosmochimica Acta* **199**, 196-209 (2017).

2 Klaver, M., Ionov, D. A., Takazawa, E. & Elliott, T. The non-chondritic Ni isotope composition of Earth’s mantle. *Geochimica et Cosmochimica Acta* **268**, 405-421 (2020).

3 Lyubetskaya, T. & Korenaga, J. Chemical composition of Earth's primitive mantle and its variance: 1. Method and results. *Journal of Geophysical Research: Solid Earth* **112** (2007).

4 Herzberg, C. *et al.* Nickel and helium evidence for melt above the core–mantle boundary. *Nature* **493**, 393 (2013).

5 Wang, S.-J., Rudnick, R. L., Gaschnig, R. M., Wang, H. & Wasylenki, L. E. Methanogenesis sustained by sulfide weathering during the Great Oxidation Event. *Nature Geoscience* **12**, 296-300, doi:10.1038/s41561-019-0320-z (2019).

6 Cameron, V., Vance, D., Archer, C. & House, C. H. A biomarker based on the stable isotopes of nickel. *Proceedings of the National Academy of Sciences* **106**, 10944-10948 (2009).

7 Gueguen, B., Rouxel, O., Ponzevera, E., Bekker, A. & Fouquet, Y. Nickel Isotope Variations in Terrestrial Silicate Rocks and Geological Reference Materials Measured by MC‐ICP‐MS. *Geostandards and Geoanalytical Research* **37**, 297-317 (2013).

8 Urey, H. C. The thermodynamic properties of isotopic substances. *Journal of the Chemical Society (Resumed)*, 562-581 (1947).

9 Richet, P., Bottinga, Y. & Javoy, M. A review of hydrogen, carbon, nitrogen, oxygen, sulphur, and chlorine stable isotope fractionation among gaseous molecules. *Annual Review of Earth and Planetary Sciences* **5**, 65-110 (1977).

10 Bostroem, D. Single-crystal X-ray diffraction studies of synthetic Ni-Mg olivine solid solutions. *American Mineralogist* **72**, 965-972 (1987).

11 Wang, W. *et al.* Concentration effect on equilibrium fractionation of Mg-Ca isotopes in carbonate minerals: Insights from first-principles calculations. *Geochimica et Cosmochimica Acta* **208**, 185-197 (2017).

12 Li, Y., Wang, W., Huang, S., Wang, K. & Wu, Z. First-principles investigation of the concentration effect on equilibrium fractionation of K isotopes in feldspars. *Geochimica et Cosmochimica Acta* **245**, 374-384 (2019).

13 Wang, W. *et al.* Effect of Ca content on equilibrium Ca isotope fractionation between orthopyroxene and clinopyroxene. *Geochimica et Cosmochimica Acta* **219**, 44-56 (2017).

14 Feng, C., Qin, T., Huang, S., Wu, Z. & Huang, F. First-principles investigations of equilibrium calcium isotope fractionation between clinopyroxene and Ca-doped orthopyroxene. *Geochimica et Cosmochimica Acta* **143**, 132-142 (2014).

15 Wu, Z., Huang, F. & Huang, S. Isotope fractionation induced by phase transformation: First-principles investigation for Mg2SiO4. *Earth and Planetary Science Letters* **409**, 339-347, doi:<https://doi.org/10.1016/j.epsl.2014.11.004> (2015).

16 Schauble, E., Rossman, G. R. & Taylor Jr, H. P. Theoretical estimates of equilibrium chromium-isotope fractionations. *Chemical Geology* **205**, 99-114 (2004).

17 Hill, P. S. & Schauble, E. A. Modeling the effects of bond environment on equilibrium iron isotope fractionation in ferric aquo-chloro complexes. *Geochimica et Cosmochimica Acta* **72**, 1939-1958 (2008).

18 Young, E., Tonui, E., Manning, C., Schauble, E. & Macris, C. Spinel–olivine magnesium isotope thermometry in the mantle and implications for the Mg isotopic composition of Earth. *Earth and Planetary Science Letters* **288**, 524-533 (2009).

19 Dauphas, N. *et al.* A general moment NRIXS approach to the determination of equilibrium Fe isotopic fractionation factors: application to goethite and jarosite. *Geochimica et Cosmochimica Acta* **94**, 254-275 (2012).

20 Zhang, Y. *Geochemical kinetics*. (Princeton University Press, 2008).

21 Richter, F. M., Liang, Y. & Davis, A. M. Isotope fractionation by diffusion in molten oxides. *Geochimica et Cosmochimica Acta* **63**, 2853-2861 (1999).

22 Watson, H. C., Richter, F., Liu, A. & Huss, G. R. Iron and nickel isotope fractionation by diffusion, with applications to iron meteorites. *Earth Planetary Science Letters* **451**, 159-167 (2016).

23 Oeser, M., Dohmen, R., Horn, I., Schuth, S. & Weyer, S. Processes and time scales of magmatic evolution as revealed by Fe–Mg chemical and isotopic zoning in natural olivines. *Geochimica et Cosmochimica Acta* **154**, 130-150 (2015).

24 Rubie, D. C. *et al.* Heterogeneous accretion, composition and core–mantle differentiation of the Earth. *Earth Planetary Science Letters* **301**, 31-42 (2011).

25 Braukmüller, N., Wombacher, F., Funk, C. & Münker, C. Earth’s volatile element depletion pattern inherited from a carbonaceous chondrite-like source. *Nature geoscience* **12**, 564-568 (2019).

26 Fischer-Gödde, M. *et al.* Ruthenium isotope vestige of Earth’s pre-late-veneer mantle preserved in Archaean rocks. *Nature* **579**, 240-244 (2020).

27 Varas-Reus, M. I., König, S., Yierpan, A., Lorand, J.-P. & Schoenberg, R. Selenium isotopes as tracers of a late volatile contribution to Earth from the outer Solar System. *Nature geoscience* **12**, 779-782 (2019).

28 Wang, Z. & Becker, H. Ratios of S, Se and Te in the silicate Earth require a volatile-rich late veneer. *Nature* **499**, 328-331 (2013).

29 Chernonozhkin, S. M., Goderis, S., Lobo, L., Claeys, P. & Vanhaecke, F. Development of an isolation procedure and MC-ICP-MS measurement protocol for the study of stable isotope ratio variations of nickel. *Journal of Analytical Atomic Spectrometry* **30**, 1518-1530 (2015).

30 Gall, L., Williams, H., Siebert, C. & Halliday, A. Determination of mass-dependent variations in nickel isotope compositions using double spiking and MC-ICPMS. *Journal of Analytical Atomic Spectrometry* **27**, 137-145 (2012).

31 Estrade, N. *et al.* Weathering and vegetation controls on nickel isotope fractionation in surface ultramafic environments (Albania). *Earth and Planetary Science Letters* **423**, 24-35 (2015).

32 Steele, R. C., Elliott, T., Coath, C. D. & Regelous, M. Confirmation of mass-independent Ni isotopic variability in iron meteorites. *Geochimica et Cosmochimica Acta* **75**, 7906-7925 (2011).

33 Gall, L. *et al.* Nickel isotopic compositions of ferromanganese crusts and the constancy of deep ocean inputs and continental weathering effects over the Cenozoic. *Earth and Planetary Science Letters* **375**, 148-155 (2013).

34 Ratié, G. *et al.* Nickel isotope fractionation during tropical weathering of ultramafic rocks. *Chemical Geology* **402**, 68-76 (2015).

35 Cameron, V. & Vance, D. Heavy nickel isotope compositions in rivers and the oceans. *Geochimica et Cosmochimica Acta* **128**, 195-211 (2014).

36 Wang, S.-J. & Wasylenki, L. E. Experimental constraints on reconstruction of Archean seawater Ni isotopic composition from banded iron formations. *Geochimica et Cosmochimica Acta* **206**, 137-150, doi:<http://doi.org/10.1016/j.gca.2017.02.023> (2017).

37 Dauphas, N. The isotopic nature of the Earth’s accreting material through time. *Nature* **541**, 521 (2017).

38 Nanne, J. A., Nimmo, F., Cuzzi, J. N. & Kleine, T. Origin of the non-carbonaceous–carbonaceous meteorite dichotomy. *Earth and Planetary Science Letters* **511**, 44-54 (2019).

39 Regelous, M., Elliott, T. & Coath, C. D. Nickel isotope heterogeneity in the early Solar System. *Earth Planetary Science Letters* **272**, 330-338 (2008).

40 Steele, R. C., Coath, C. D., Regelous, M., Russell, S. & Elliott, T. Neutron-poor nickel isotope anomalies in meteorites. *The Astrophysical Journal* **758**, 59 (2012).

41 Tang, H. & Dauphas, N. Abundance, distribution, and origin of 60Fe in the solar protoplanetary disk. *Earth and Planetary Science Letters* **359**, 248-263 (2012).

42 Tang, H. & Dauphas, N. 60Fe–60Ni chronology of core formation in Mars. *Earth and Planetary Science Letters* **390**, 264-274 (2014).

43 Chernonozhkin, S. M., Goderis, S., Costas-Rodríguez, M., Claeys, P. & Vanhaecke, F. Effect of parent body evolution on equilibrium and kinetic isotope fractionation: a combined Ni and Fe isotope study of iron and stony-iron meteorites. *Geochimica et Cosmochimica Acta* **186**, 168-188 (2016).

44 Wu, F.-Y., Walker, R. J., Yang, Y.-H., Yuan, H.-L. & Yang, J.-H. The chemical-temporal evolution of lithospheric mantle underlying the North China Craton. *Geochimica et Cosmochimica Acta* **70**, 5013-5034 (2006).

45 Liu, J. *et al.* Processes controlling highly siderophile element fractionations in xenolithic peridotites and their influence on Os isotopes. *Earth and Planetary Science Letters* **297**, 287-297 (2010).

46 Rudnick, R. L., Gao, S., Ling, W.-l., Liu, Y.-s. & McDonough, W. F. Petrology and geochemistry of spinel peridotite xenoliths from Hannuoba and Qixia, North China craton. *Lithos* **77**, 609-637 (2004).

47 Xu, Y.-G. *et al.* Late Archean to Early Proterozoic lithospheric mantle beneath the western North China craton: Sr–Nd–Os isotopes of peridotite xenoliths from Yangyuan and Fansi. *Lithos* **102**, 25-42 (2008).

48 Liu, J. *et al.* Comparative Sr–Nd–Hf–Os–Pb isotope systematics of xenolithic peridotites from Yangyuan, North China Craton: additional evidence for a Paleoproterozoic age. *Chemical Geology* **332**, 1-14 (2012).

49 Fabriès, J., Lorand, J.-P. & Bodinier, J.-L. J. T. Petrogenetic evolution of orogenic lherzolite massifs in the central and western Pyrenees. **292**, 145-167 (1998).

50 Maier, W. D. *et al.* Progressive mixing of meteoritic veneer into the early Earth’s deep mantle. *Nature* **460**, 620 (2009).

51 Walker, R. J., Shirey, S. B. & Stecher, O. Comparative ReOs, SmNd and RbSr isotope and trace element systematics for Archean komatiite flows from Munro Township, Abitibi Belt, Ontario. *Earth and Planetary Science Letters* **87**, 1-12 (1988).

52 Helz, R. T. Differentiation behavior of Kilauea Iki lava lake, Kilauea Volcano, Hawaii: an overview of past and current work. *Magmatic processes: physicochemical principles* **1**, 241-258 (1987).

53 Liu, S.-A. *et al.* Copper isotopic composition of the silicate Earth. *Earth and Planetary Science Letters* **427**, 95-103 (2015).

54 Li, W.-Y. *et al.* Empirical calibration of the clinopyroxene–garnet magnesium isotope geothermometer and implications. *Contributions to Mineralogy and Petrology* **171**, 61 (2016).

55 Wang, S.-J. *et al.* Tracing subduction zone fluid-rock interactions using trace element and Mg-Sr-Nd isotopes. *Lithos* **290**, 94-103 (2017).
